# Tuning the Wettability of Hydrophobic Metal–Organic Frameworks by Linker-Doping

**DOI:** 10.1021/acsnano.5c18554

**Published:** 2026-05-04

**Authors:** Davide Caporale, Daniel Moreno-Rodríguez, Andrea Le Donne, Eder Amayuelas, Luis Bartolomé, Cléophée Gourmand, Gabriel Alejandro López, Kerman Gómez, Juan Miguel López del Amo, Antonio Tinti, Sebastiano Merchiori, Alberto Giacomello, Simone Meloni, Yaroslav Grosu

**Affiliations:** † 138819 Centre for Cooperative Research on Alternative Energies (CIC EnergiGUNE), Basque Research and Technology Alliance (BRTA), Alava Technology Park, Albert Einstein 48, Vitoria-Gasteiz 01510, Spain; ‡ Department of Physics, 16402 University of the Basque Country (UPV/EHU), Bilbao 48490, Spain; § Dipartimento di Ingegneria Meccanica e Aerospaziale, Sapienza Università di Roma, Rome 00184, Italy; ∥ Dipartimento di Scienze Chimiche, 9299 Università degli Studi di Ferrara, Farmaceutiche e Agrarie, Ferrara 44121, Italy; ⊥ Laboratory of Molecular Simulation (LSMO), 27218 Institut des Sciences et Ingénierie Chimiques and Ecole Polytechnique Fédérale de Lausanne (EPFL), Rue de l’Industrie 17, Sion 1951, Switzerland; # Institute of Chemistry, 49568 University of Silesia, Katowice 40-006, Poland

**Keywords:** metal organic frameworks, ZIF-7–8, water intrusion-extrusion, hydrogen-bond, non-local wetting phenomena, energy storage

## Abstract

The wetting behavior of hydrophobic nanoporous materials plays a pivotal role in advanced technologies such as energy storage, molecular separations, catalysis, and biomimetic systems. A central challenge in employing these materials is the precise control of wetting (intrusion) and dewetting (extrusion) pressures. To address this, we investigated how varying the concentration of organic linkers in ZIF-7–8 (composed of Zn-methylimidazole and benzimidazole) influences the intrusion-extrusion behavior of water within its micropores. Remarkably, we noticed that even a minor substitution of organic linkers (below 3%) led to significant and systematic changes in the wetting properties. Owing to the small fraction of substituted linkers, such effect could not be explained by classical models that consider individual cages. To elucidate this phenomenon, we combined experiments with molecular dynamics simulations and stochastic modeling of the crystallites. Our results reveal that the introduction of alien linkers perturbs the hydrogen-bond network, thereby disrupting the cooperative effects fundamental to the intrusion-extrusion process.

## Graphical abstract

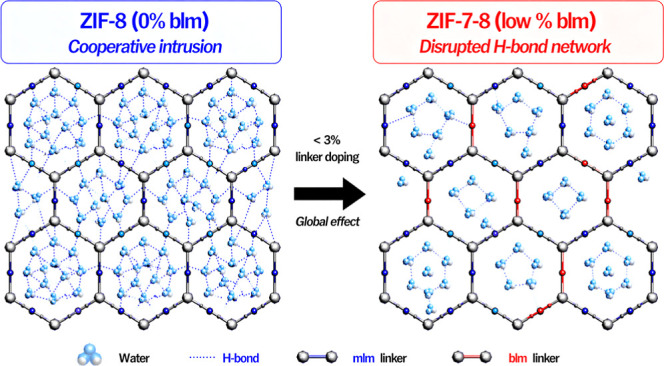

The wetting behavior of hydrophobic nanoporous materials is a central factor in a range of advanced technologies, from energy storage and molecular separations to catalysis and biomimetic systems. In particular, the ability to control wetting at the nanoscale is essential in membrane-based filtration systems, where hydrophobic nanopores can be used to prevent spontaneous water flow, thereby enabling selective molecular transport only under externally applied pressures. This is particularly relevant in membrane distillation, desalination, and pressure-driven nanofiltration, where pore wetting directly affects separation efficiency and fouling resistance.^1–5^ In the field of catalysis, nanoporous materials such as hydrophobic zeolites and metal–organic frameworks (MOFs) provide confined reaction environments that can be modulated by the presence or absence of liquid phases. The degree of wetting influences reactant diffusion, active site accessibility, and even the local dielectric environment, which in turn affects reaction kinetics and selectivity.^6–10^ Biological systems, such as some ion channels, also exploit the controlled wetting/dewetting of their pore to switch on/off the ionic currents across the plasma membrane by a process known as hydrophobic gating.^11–13^ Such precise regulation of ion and water transport by wetting/dewetting has inspired the design of hydrophobically gated fluidic memristors based on engineered biological nanopores.^14,15^ The forced intrusion of nonwetting liquids such as water into hydrophobic nanopores enables the storage and dissipation of mechanical energy, which has been explored for applications like molecular springs, thermal actuators, and shock absorbers.^16–23^ Finally, nanoconfinement can significantly reduce the critical temperature of liquids,^24–27^ with hydrophobic environments proving especially effective, lowering the critical temperature of water by up to 250 K.^28^

One of the central difficulties of the aforementioned applications is precise control of intrusion (wetting) and extrusion (dewetting) pressures, as well as the difference between them (hysteresis).

When the pore size approaches the molecular dimension of the liquid, the validity of classical capillarity becomes strongly system dependent. For highly nanoconfined nonwetting liquid the classical capillarity descriptions breakdown.^29^ While recent studies have shown that macroscopic formulations such as the Kelvin equation can remain applicable down to angstrom-scale confinement in atomically smooth wetting systems with mechanically compliant walls,^30^ such compensating effects are absent in a nonwetting regime. Furthermore, secondary pore connectivity and framework flexibility introduce additional variables that modify wetting behavior. Even angstrom-scale interconnections between pores can shift intrusion pressures and affect the thermodynamics of phase transitions within the material.^31,32^

Recent works have demonstrated that water intrusion in hydrophobic MOFs can be governed by collective nonlocal effects rather than by independent pore filling. In ZIF type frameworks, cooperative intrusion and extrusion are sustained by hydrogen-bond networks propagating across intercage apertures via an avalanche-like wetting front.^33,34^ This cooperativity has been shown to depend strongly on framework flexibility, crystallite size, and pore connectivity.^18,35,36^ In parallel, defect-induced wetting transitions have been reported, where structural or chemical heterogeneities fragment wetting fronts, thereby modifying intrusion pressures and hysteresis.^29,37^ These findings establish that wetting in hydrophobic MOFs results from the interplay between local energetics and long-range hydrogen bonds, and that even localized perturbations can play a role on macroscopic intrusion-extrusion behavior.

In this work, we explore linker doping as a strategy to control the water-wettability of a hydrophobic MOF by partially substituting the original linkers with ones of a different chemical nature. Unexpectedly, we found that even at extremely low concentrations, where only some of the cages contain the second linker (less than one modified linker per cage), the overall wettability of the entire framework changes significantly. This surprising behavior cannot be explained by conventional arguments on individual pores, such as changes in their size, topology, or local hydrophobicity, as the fraction of substituted linkers is too small to directly affect each pore in this way. The effect of linker substitution and pore geometry on the wettability of MOFs has been widely investigated,^38,39^ but the impact of dilute linker substitution on collective wetting behavior has remained largely unexplored.

Using a combination of experiments, molecular dynamics simulations, and stochastic modeling, we show that the introduction of a chemically distinct linker sizably changes the characteristics of intruded water in nearby pristine cages. This change alters the energy landscape of neighboring unmodified cages, giving rise to a nonlocal cooperative effect. These findings challenge the current understanding of wettability in nanoporous materials and open new avenues for tuning intrusion and extrusion pressures in applications such as filtration, separation, chromatography, catalysis, ionic transport, and energy storage and conversion.

## Results/Discussion

### Experiments

ZIF-8 and ZIF-7 both belong to the family of Zeolitic Imidazolate Frameworks (ZIFs), sharing the same sodalite (SOD) topology. Their structures consist of polyhedral cages interconnected by six-membered ring (6MR) apertures—Figure 1. The two frameworks differ only in linker chemistry: ZIF-8 is built from 2-methylimidazolate (mIm) organic linkers, whereas ZIF-7 contains the bulkier benzimidazolate linkers (bIm).^40^ Their intrusion-extrusion characteristics differ significantly. Pure ZIF-8 is known to exhibit water intrusion-extrusion with moderate hysteresis. In contrast, ZIF-7, which contains only bIm linkers, displays significantly higher intrusion pressures, chemical instability in water and reduced pore accessibility due to steric hindrance at the 6MR apertures.^35^

**Figure 1:**
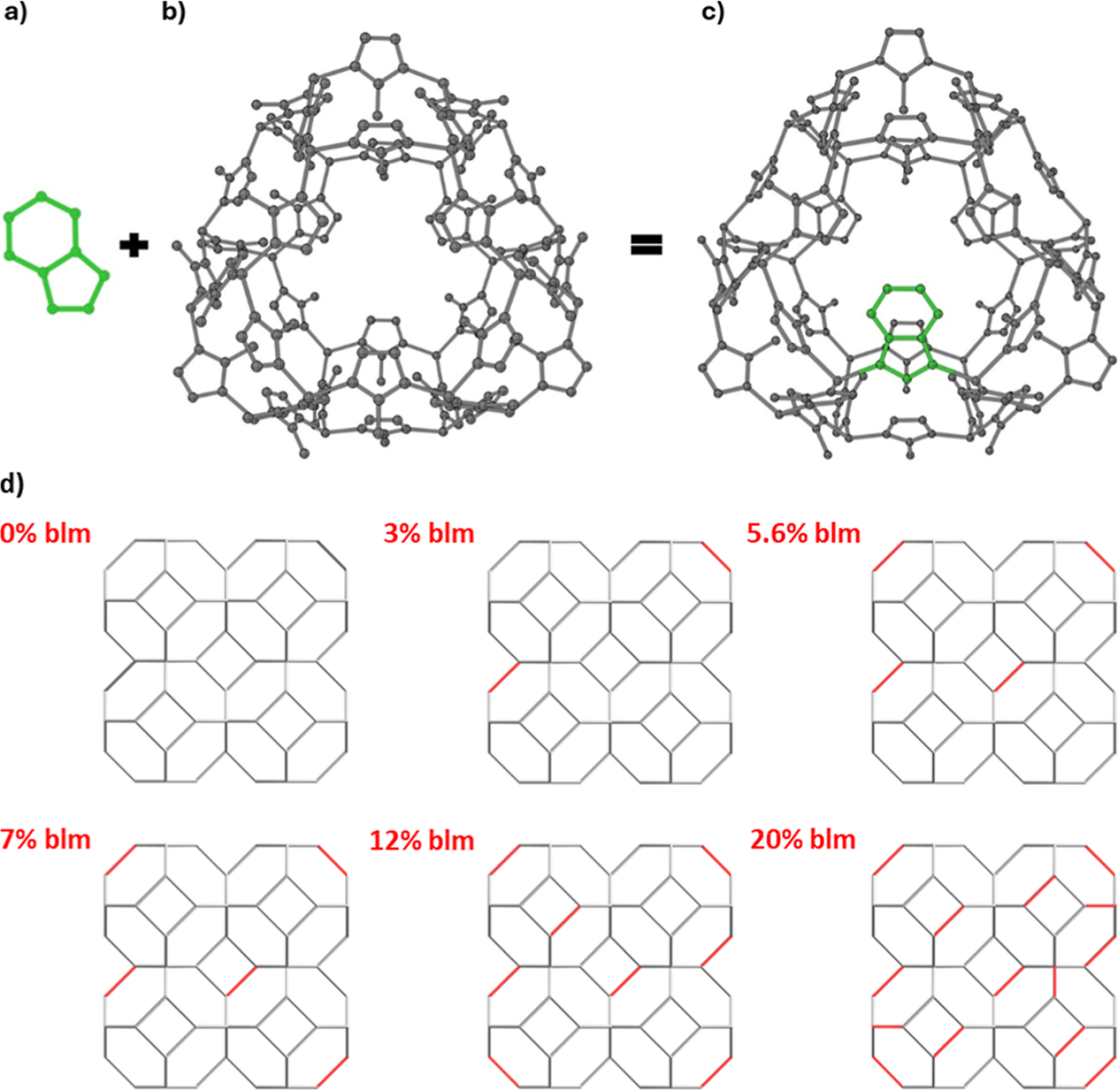
(a) Benzimidazole molecule, (b) ZIF-8, and (c) ZIF-7–8 single cage; (d) topological model of ZIF-7–8 with different concentrations of blm (red color). Note that below 3% blm not all the cages of ZIF-7–8 contain blm linker even under the assumption of homogeneous distribution. By replacing mIm linkers with bIm, we obtain a hybridized structure while maintaining the same topology.

To investigate the wettability of mixed-linker hydrophobic MOFs, we synthesized a series of hybrid ZIF-7-8 MOFs (Section SI1) by substituting some of the mIm linkers in ZIF-8 with bIm linkers from ZIF-7 (Figure 1, Table SI1). All the samples consist of macroscopic crystallites (Section SI9); therefore, differences in their size are not expected to influence the wetting characteristics, an effect that has only been observed for crystallite sizes below 100 nm.^34^

The concentration of bIm was verified by NMR in the 0–29% range of bIm concentration (Section SI3, Figure SI1). X-ray diffraction (XRD) confirmed that this substitution preserves the SOD topology^41–43^ and cage structure, with only minor distortions (Section SI4, Figures SI2 and SI12). This concentration range was selected because ZIF-7-8 crystallizes only within two distinct regions: 0–35% and 80–100% of bIm.^44^ While the ZIF-8 cage structure can be affected by applied pressure,^45^ at the experimental pressures considered here no sizable structural changes are expected that would alter our discussion.^46^

Nitrogen adsorption–desorption at 77 K, further validate preservation of microporosity while revealing modifications of the framework flexibility upon linker substitution (Section SI5, Figures SI13–SI17). The low-pressure region (*P*/*P*_0_ ≤ 0.04) of the isotherms is shown in the magnified view in Figure 2a. In agreement with literature,^47,48^ the undoped samples (ZIF-8) show 3 clear adsorption steps, with the second uptake centered at *P*/*P*_0_ ∼ 0.005. This behavior originates from reversible reorientation of the mIm linkers, which increases the effective aperture size known as gate-opening effect.^50,59^ Upon increasing the bIm content, the step at *P*/*P*_0_ ∼ 0.005 progressively weakens being completely undetectable above 9% while the overall microporous character of the isotherm is retained. The suppression of this step indicates that the bulkier benzimidazole linker modifies the adsorption-induced flexibility response of the framework. This is in line with gas adsorption experiments performed on ZIF-7.^44,49^ Therefore, the modification of the gate-opening can serve as indicator of cages that poses a sufficient number of mlm to demonstrate this flexibility effect.

**Figure 2:**
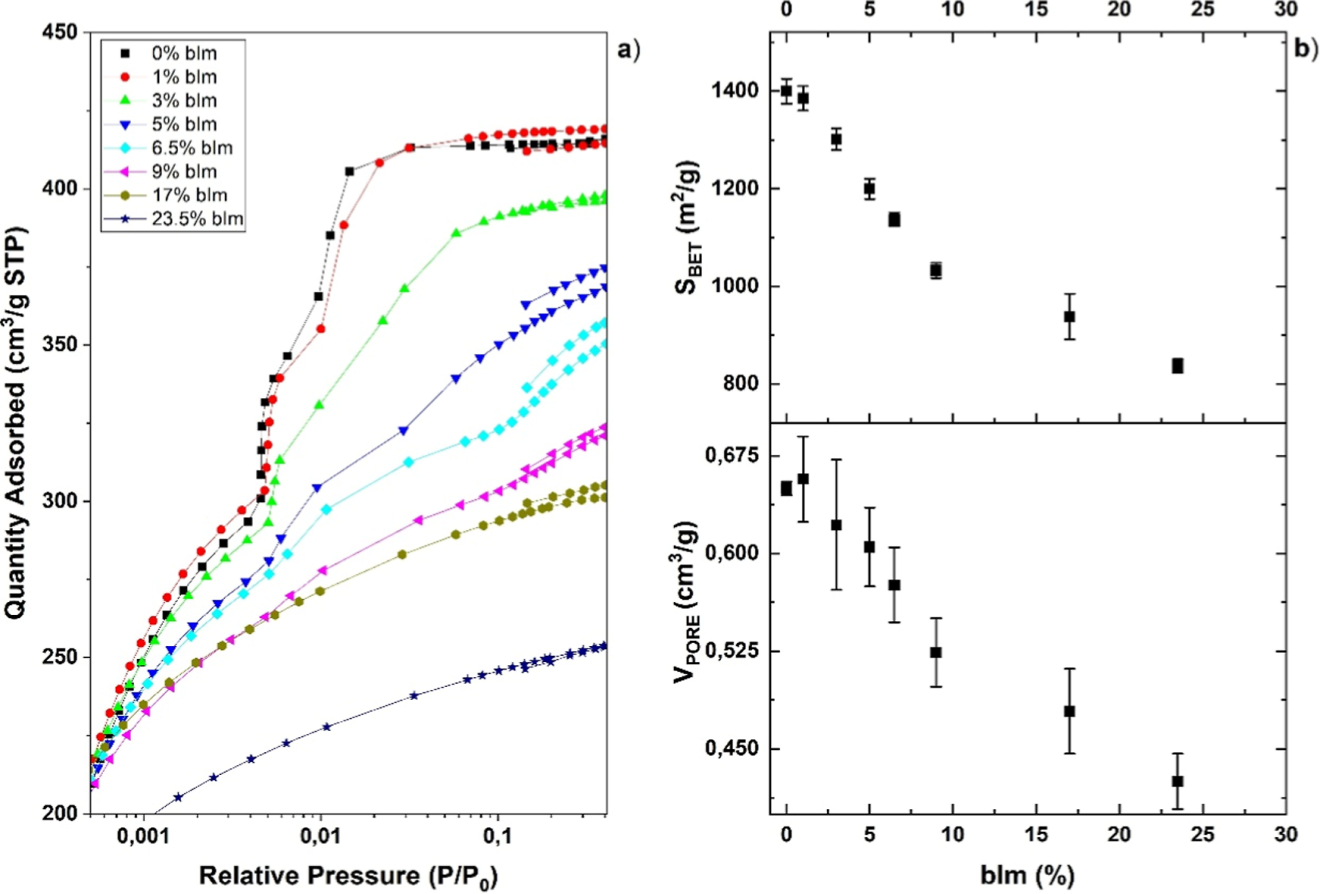
(a) N_2_ adsorption isotherms at 77K of ZIF-7-8 samples of different bIm concentration. (b) Dependence of surface area (*S*_BET_) and pore volume (V_PORE_) on bIm content.

High-pressure water intrusion–extrusion isotherms for these MOFs are shown in Figure 3a. All key parameters of the wetting–dewetting process, namely, intrusion/extrusion pressure (*P*_int_ and *P*_ext_), volume, and hysteresis, exhibit a nonlinear yet monotonic dependence on bIm concentration (Figure 3b). *P*_int_ and *P*_ext_ were defined as the pressures corresponding to the maxima of the dV/dP derivative of the intrusion and extrusion PV isotherms. This criterion avoids ambiguities associated with arbitrary choices and is commonly used for nonideal transitions. Both *P*_int_ and *P*_ext_ increase with increasing bIm concentration. The hysteresis, defined as the difference between intrusion and extrusion pressures, decreases with increasing bIm concentration (Figure 3b), as does the intruded volume.

**Figure 3:**
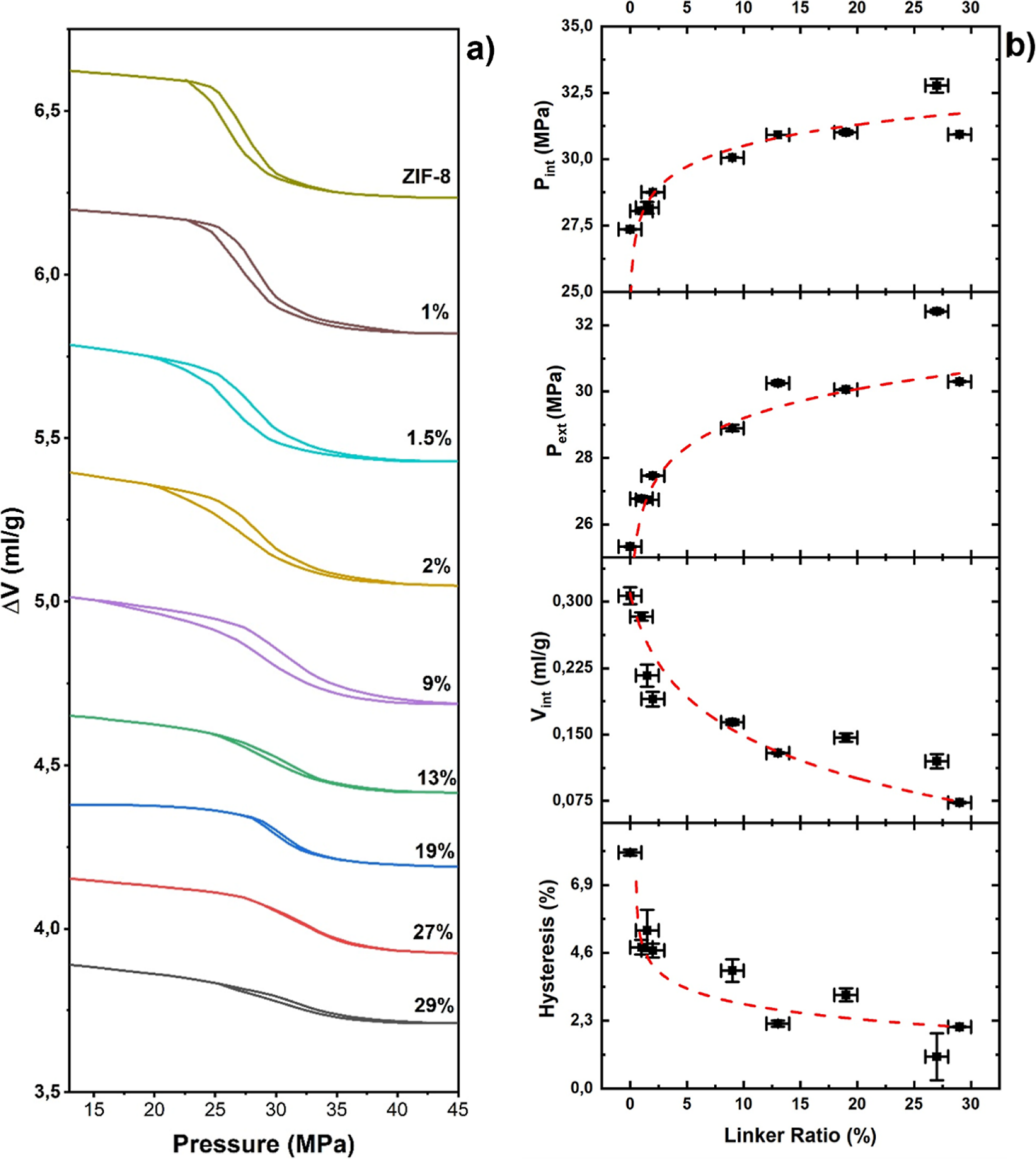
(a) Experimental *PV*-isotherms of water intrusion-extrusion cycles within ZIF 7-8 at varying bIm concentrations. (b) Dependence of water intrusion/extrusion pressure, intrusion volume, and hysteresis on bIm content.

Remarkably, all key wetting–dewetting parameters show a sizable dependence on bIm content even at nominal concentrations below one bIm linker per cage (∼3%) (see Section SI6, Table SI2 and Figure SI18). For example, an increase of ∼1.3 MPa in intrusion pressure is observed for a sample at 3% bIm concentration. A 1.3 MPa shift in operating pressure is technologically meaningful. Furthermore, the intrusion–extrusion process occurs in a single step, despite the coexistence of pure ZIF-8 cages (containing only mIm linkers) and mixed-linker cages (containing both mIm and bIm linkers). This behavior suggests a collective, nonlocal effect of bIm doping that modifies the wettability of the entire framework, including undoped ZIF-8 cages.

Importantly, the 3% value is only a nominal threshold for having one bIm linker per cage under the assumption of a uniform distribution of minority linkers. Any deviation from uniformity, where some cages contain more than one bIm linker, would necessarily leave other cages as pure ZIF-8. Thus, if the minority-linker distribution is nonuniform (as supported by our microscopic analysis in Section SI10 of the Supporting Information), concentrations higher than 3% are required to ensure at least one bIm per cage. This further supports our hypothesis of a collective, nonlocal effect of bIm doping on framework wettability. We provide additional evidence and a mechanistic explanation for this behavior below, based on a stochastic wetting/drying model of mixed-linker ZIF-7-8 metal–organic framework crystallites.

In summary, the fact that the intrusion–extrusion characteristics change significantly at bIm concentrations below 3% (the nominal threshold for one bIm per cage under a homogeneous distribution) indicates that bIm-containing cages affect the wettability of nearby bIm-free cages through cooperative, nonlocal interactions. Moreover, if the minority linkers are distributed nonuniformly, a possibility supported by our microscopic analysis discussed in the Supporting Information, the concentration required for all cages to contain at least one bIm linker is higher than 3%. Therefore, also the changes in the intrusion–extrusion parameters observed at nominal bIm concentrations above 3% cannot be attributed solely to local compositional changes, further reinforcing the hypothesis of a cooperative, nonlocal influence of bIm-containing cages on the wetting/drying behavior of the entire crystallite. This extends the relevance of this mechanism over a broad range of nominal bIm concentrations (*x*-axis in Figure 3b).

To explain the observed phenomena, we also considered alternative, more conventional hypotheses based on the properties of metal–organic frameworks and their dependence on linker composition. Concerning pressure, we investigated whether mixing with bIm could stiffen the framework and thereby affect the intrusion pressure. To probe the effect of linker mixing on the swinging flexibility of ZIF-7-8, we performed gas adsorption experiments as a function of linker composition (Figure 2 and ref 50). For ZIF-8 and mixed-linker ZIF-7-8 with bIm contents below 9%, we observe multistep adsorption isotherms. These steps are commonly interpreted as gate-opening transitions,^50^ and their multiplicity indicates that gas adsorption in mixed-linker ZIF-7-8 is governed by local processes driven by the bIm composition of the specific cell undergoing adsorption. At higher bIm contents, the multiple steps disappear, consistent with the expectation that all (or nearly all) cells contain some bIm. In contrast to gas adsorption, liquid intrusion isotherms display a single plateau across all compositions (Figure 3a), suggesting that, for water, the process is collective, as argued above.

Regarding the effect of minority-linker content on the intruded volume, we also considered a purely geometric explanation based on steric hindrance by bulkier bIm. To evaluate the consistency of this hypothesis with the intrusion–extrusion results, we again compared with gas adsorption PV isotherms. Gas uptake also decreases with increasing bIm content; however, this reduction (∼37% at 23% bIm) is much smaller than the reduction in intruded liquid volume (∼75% at 29% bIm). More importantly, in liquid intrusion the bIm content has a strong effect already at very low concentrations, with an intruded-volume reduction of ∼30% at only 3% bIm.

Overall, the comparison between gas adsorption and liquid intrusion indicates that the effect of bulkier minority linkers cannot be explained solely in terms of local geometric constraints. Instead, cooperative, long-range interactions must be invoked. To uncover a plausible microscopic mechanism underlying this nontrivial behavior, we performed molecular dynamics simulations and stochastic modeling, as described below.

### Atomistic Simulations

To understand the atomistic origins of these effects, we investigated the wetting of a ZIF-7-8 computational sample. Starting from a 2 × 2 × 4-unit cell ZIF-8 slab, already validated in earlier studies,^33^ one mIm linker of an interior cell of the slab was replaced with one bIm (see Figure 4a). On such a system, we studied the wetting of (i) the cavity containing the bIm, denominated ZIF-7-8 cage in the following, (ii) a first neighbor ZIF-8-like cage, a cage containing no bIm connected to a ZIF-7-8 cage via a 6MR aperture, and (iii) second neighbor ZIF-8-like cage, a cage containing no bIm connected to a first neighbor ZIF-8-like cage by a 6MR aperture (Figure 4a).

**Figure 4:**
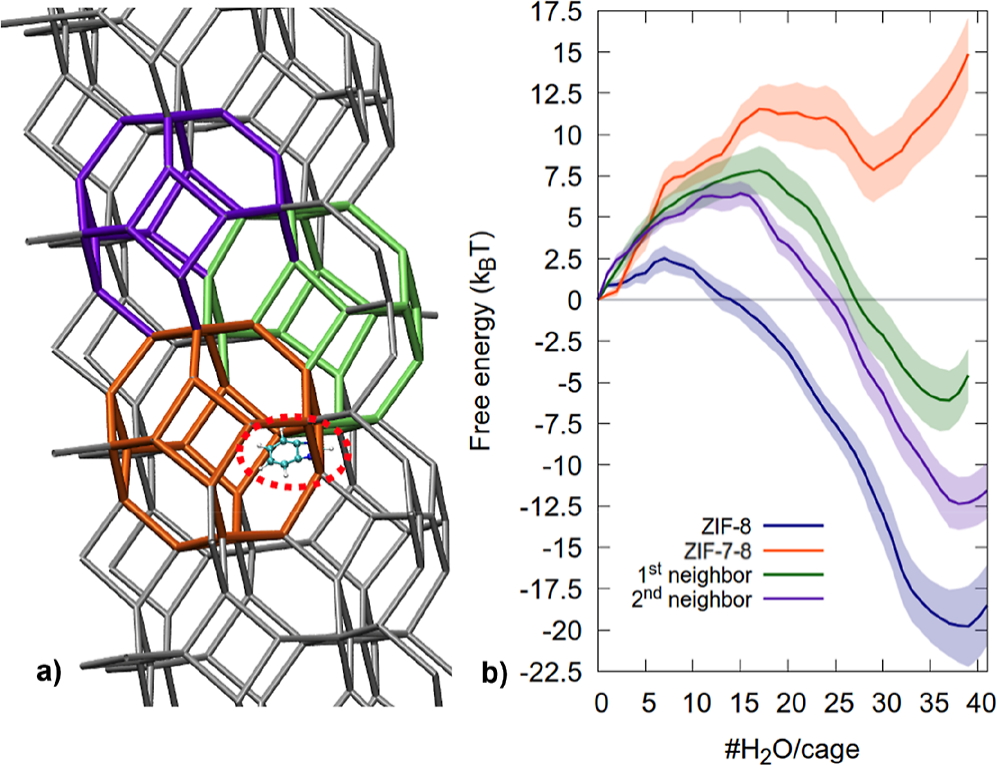
(a) A ZIF-7-8 computational model generated by starting from a ZIF-8 slab and substituting one mIm linker in an interior cell with a bIm linker (highlighted in the dashed ellipses). (b) Free energy profiles of cage filling in ZIF-8 (blue), ZIF-7-8 (orange), and 1st (green) and 2nd neighbor (violet) ZIF-8-like cages at 300 K and 25 MPa. Shaded areas indicate statistical errors.

Figure 4b compares the free energy of water intrusion at 25 MPa for the three cages described above. All cages exhibit two free energy minima corresponding to the empty and fully filled states. However, the stability and position of the wet state differ significantly. In first and second neighbor ZIF-8-like, the most stable configuration is the wet state, with an occupancy of 37 and 39 water molecules, respectively. In contrast, at the same pressure, the cage containing bIm in the ZIF-7-8 sample favors the empty state, and the filled metastable state accommodates only 29 water molecules. As a reference, in a pure ZIF-8 sample at the same thermodynamic conditions, the stable state is the wet one, which is slightly more stable than the second neighbor ZIF-8-like in the ZIF-7-8 sample. Same as in the second neighbor ZIF-8-like cage, the wet state in the pure ZIF-8 sample corresponds to 39 water molecules contained in a cage.

The free energy profiles of Figure 4b clearly indicate that the presence of bIm linkers destabilizes the intruded (wet) state in ZIF-7-8 cages and raises the intrusion energy barrier, meaning that higher pressures are required for water to wet cages of this kind. However, sizable effects are also observed in neighboring cages, which decreases with increasing distance. The difference in free energy of the wet states in first and second neighbor cells is lower than the dry state by ∼6 and ∼12.5 *k*_B_ *T*, to be compared with ∼20 *k*_B_ *T* in pure ZIF-8 (Figure 4b). Also, the intrusion barrier, the barrier the liquid must overcome for wetting a cage, is higher in first and second neighbor cells than in pure ZIF-8: ∼6 and ∼7.5 *k*_B_ *T*, respectively, vs ∼2.5 *k*_B_ *T*. At the same time, the destabilization of the wet state of ZIF-8-like cages in ZIF-7-8 samples results in a decrease of the extrusion barriers of first and second neighbor cells −13.5 and 12 *k*_B_ *T*, respectively—with respect to pure ZIF-8 (∼22.5 *k*_B_ *T*).

The destabilization of the wet state of both ZIF-7-8 and ZIF-8-like cages in ZIF-7-8 samples, the increase of the intrusion barrier and the decrease of the extrusion one result in an increase of the intrusion pressure *P*_int_ and a decrease of the extrusion pressure *P*_ext_.

Summarizing, the effect of the presence of bIm linkers directly affects the wetting of ZIF-7-8 cages and indirectly ZIF-8-like cages in the ZIF-7-8 sample, explaining the shift of the experimental *PV*-isotherms of Figure 3 to higher pressures and the reduction of hysteresis.

The atomistic origin of the changes in the free energy profiles of intrusion in ZIF-7-8 and ZIF-8-like cages, as compared to regular ZIF-8, can be explained considering the hydrogen bond bridging across 6MR apertures connecting the cages of the ZIF porous materials we considered in this work. In ZIF-7-8 cages, this effect is the result of the presence of the bIm linker that, as previously showed by Amayuelas et al.,^35^ occludes the corresponding 6MR apertures, even more than in ZIF-7 that contains only bIm linkers.

In the case of ZIF-8-like cages, this effect is indirect, since ZIF-7-8 cages contain fewer water molecules, the probability that one is close to the 6MR apertures connecting this cage to first neighbor ZIF-8 cages is lower, making the probability of forming the corresponding hydrogen bond lower, thus reducing the associated stabilization effect. A similar effect occurs in second neighbor ZIF-8 cages, due to the less water present in first neighbor ZIF-8 cages, the possibility to form hydrogen bonding across 6MR apertures connecting these cages is lower. To test this hypothesis, we measured the number of hydrogen bonds formed by water molecules lying within 2.5 Å from the center of the 6MR apertures between ZIF-7-8 and first neighbor ZIF-8 cages, between first neighbor ZIF-8 and second neighbor ZIF-8 cages, and in pure ZIF-8. The average number of hydrogen bonds passes from ∼0.5 in the first case to ∼0.8 in the second, the latter almost corresponding to the value measured in pure ZIF-8, ∼0.83.

### Stochastic Network Model

After revealing the atomic mechanism by which the presence of a single bIm alters the wetting of neighboring cages, we ask ourselves what the effect of this local change on the overall wetting of a crystallite is. To compute the intrusion and extrusion cycle on this relatively large scale, we develop a stochastic model based on that of,^33^ in which each cage has only two states: wet or dry. The state of each cage is then updated based on the wetting/drying probability that depends on external parameters, e.g., pressure, on the nature of the cage (ZIF-8 or ZIF-7-8), and on the state of neighboring cages. These interactions are modeled based on the molecular dynamics results in the previous section, while the pressure is changed according to the experimental protocol. Overall, this model bridges atomistic insights and experiments at a qualitative level, serving as a minimal mechanistic framework to assess whether disruption of hydrogen-bond-mediated intercage cooperativity by a dopant linker is sufficient to reproduce the experimentally observed collective effect of linker doping wetting behavior.

The stochastic model of the crystallite was constructed with an SOD topology, reflecting that of ZIF-8 and ZIF-7-8 within the bIm concentration range explored in this work. The model contains two types of cages: A (mimicking ZIF-8 cages made exclusively of mIm linkers) and B (mimicking ZIF-7-8 cages with bIm linkers). Each cage is connected to eight nearest neighbors via 6MR gates (Figure SI19).^34^ The probability of each cage transitioning between dry and wet states depends on the wetting and drying free-energy barriers (ω_w_ and ω_d_) and on the wet/dry cage volumes (*v*_w_ and *v*_d_), all depend on the cage nature (A or B), and on the applied pressure *P*. Additionally, in ref 33 we have demonstrated that the wetting free-energy profile of a cage depends on the wetting state of neighboring ones: if neighboring cages are wet, hydrogen bonds can form across 6MR apertures, favoring wetting of the considered cage. The presence of a bulkier linker, such as bIm, makes hydrogen bonding across two cages—and therefore wetting—harder, see the previous section. This nonlocal wetting effect is accounted for in the model by different intercage interaction coefficients (*I*_AA_, *I*_AB_, *I*_BA_, *I*_BB_) that modulate the wetting probability depending on the state and nature of the neighboring cages. To reflect the atomistic results of Figure 4, sharp contrasts in intercage interaction strengths are introduced: strong A–A coupling (*I*_AA_ = 0.95), moderate cross-type coupling (*I*_AB_ = *I*_BA_ = 0.5), and very weak B–B interactions (*I*_BB_ = 0.01). Overall, the effective wetting and drying free-energy barriers are defined as

$$\Omega_{w}^{i}=\left(\omega_{w}^{i}-P\cdot v_{w}^{i}+\left(\frac{n_{\max}}{2}-n\right)\cdot I\right)$$

$$\Omega_{d}^{i}=\left(\omega_{d}^{i}+P\cdot v_{d}^{i}+\left(n-\frac{n_{\max}}{2}\right)\cdot I\right)$$

where the index i determines the cage type (A or B), *n*_max_ and *n* denote the maximum and current number of wet neighbors, respectively, and the interaction parameter *I* captures the effect of hydrogen bonds across cages

$$I=\frac{n_{A}\left(n_{A}I_{AA}+n_{B}I_{AB}\right)+n_{B}\left(n_{A}I_{BA}+n_{B}I_{BB}\right)}{n_{\max}^{2}}$$

with *n*_A_ and *n*_B_ the number of neighbors of A- and B-type cages, respectively. Although rather simplified, this model captures the main experimental trends; parameters used below were chosen to approximately map the experimental bIm concentration into B-cage content. Further details on the chosen parameters and on the sensitivity of the results to their value are provided in Supporting Information, Section SI8.

We systematically explored the influence of bIm linker content by increasing fractions of B-type cages in the model. Figure 5 shows that even minimal incorporation of B cages (<5%) leads to a significant increase in both *P*_int_ and *P*_ext_ and to the decrease of hysteresis relative to the case with only A cages (Figure 5a–c and Figures SI20–S25), in agreement with experimental observations (Figure 3b). In the latter case, it is easier to wet individual cages as shown by MD simulations and encoded by ω_w_^A^; in addition, once an A-type cage is wet, the strong A–A interactions facilitate the wetting of its neighbors. This triggers the wetting of the entire crystallite by an abrupt process which occurs via a coherent liquid front that encloses a single dry domain (“bubble”) which disappears at a well-defined pressure (Figure SI26).^33^ Similarly, dewetting occurs through the abrupt formation of a single bubble that starts from a random seed within the bulk domain.^33^ However, as B-type cages are introduced in the ZIF-7-8 model, the intruding liquid front encounters cages that are both harder to wet (ω_w_^B^ > ω_w_^A^) and less connected with their neighbors (*I*_AB_ = *I*_BA_ = 0.5, *I*_BB_ = 0.01). Effectively, they behave as wetting defects that pin^37^ and fragment the liquid front. Indeed, both the intrusion and extrusion processes are characterized by a less compact liquid front (Figure 5d–e, Figures SI28 and SI29) and by the presence of multiple, smaller bubbles that do not completely disappear even at the highest pressures (Figure SI26). Wetting defects hinder the intrusion process, leading to an increase in the intrusion pressure. On the other hand, the presence of multiple B-type seeds for bubble nucleation (with ω_d_^B^ > ω_d_^A^) facilitates extrusion, leading to an increase in *P*_ext_ (Figures S19–SI24). Results show that the increase in *P*_ext_ is larger than that in *P*_int_, leading to a significant reduction of the hysteresis down to almost zero area, i.e., to a reversible *PV*-cycle. This is likely due to the presence of pre-existing nuclei that allow the system to bypass the nucleation of a new bubble from the metastable liquid (Figure SI26).

**Figure 5:**
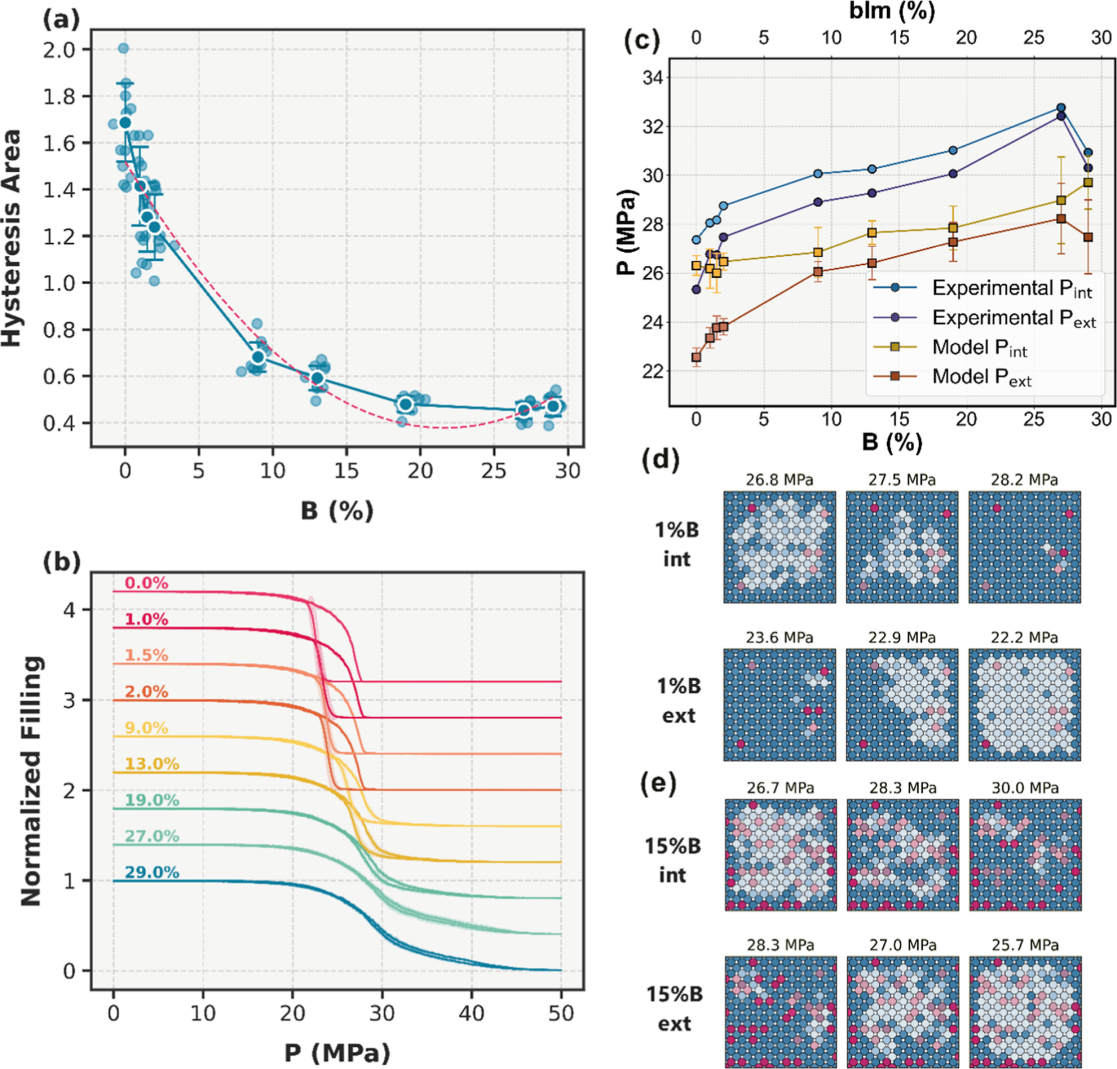
Influence of B-type cages content on the pressure-responsive behavior of ZIF-7-8 in the Stochastic model. (a) Dependence of the hysteresis area on the B-type cage content (B%). (b) Normalized filling curves highlighting the change in the steepness of the intrusion step with varying B-type cages content. (c) Comparison between experimental and simulated intrusion (*P*_int_) and extrusion (*P*_ext_) pressures as a function of B-type cages content, demonstrating good agreement between the model and the experimental data. Snapshots of a crystallite model section showing the spatial distribution of filled (blue) and empty (pale blue) A cages, and of filled (pink) and empty (pale pink) B cages during the intrusion process at (d) 1% and (e) 15% B-type cages content in the system.

The interplay between local energetic barriers and collective cooperativity is further illustrated in Figure SI27, in which systematic variation of ω_w_^A^, ω_w_^B^, and *I*_AA_ and *I*_AB_ highlights their distinct roles in modulating the intrusion–extrusion behavior. Increasing ω_w_^A^ elevates both *P*_int_ and *P*_ext_ (Figure SI27b,c), reflecting the higher energy cost for intrusion and delayed extrusion of A-type cages. In contrast, higher ω_w_^B^ values mainly raise *P*_ext_ (Figure SI27c), which in B cages impede cooperative drying and broaden hysteresis (Figure SI27a). Complementarily, the coupling parameters tune the degree of cooperativity: larger *I*_AA_ values enhance collective wetting, yielding higher intrusion and extrusion pressures and wider hysteresis loops, while weaker coupling narrows the hysteresis (Figure SI27a). In turn, when *I*_AB_ is strong, B-type cages remain coupled to A-type domains, allowing cooperative transitions through the mixed crystallite. This coupling preserves a coherent pressure response, maintaining higher *P*_int_ and *P*_ext_ values (Figure SI27b,c) and moderately broad hysteresis loops characteristic of collective behavior (Figure SI27a). Conversely, weak *I*_AB_ reduces cross-domain communication, leading to fragmented switching events accompanied by lower transition pressures and narrower hysteresis (Figure SI27a). In summary, the pronounced sensitivity of wetting to small quantities of a different linker arises from the increased difficulty of wetting across 6MR apertures, which mimics the disruption of hydrogen-bonding pathways.^33^ Although bIm-containing cages exhibit higher local energy barriers for intrusion and reduced stability of the wet state compared to their mIm counterparts, the dominant factor influencing the macroscopic behavior of ZIF-7-8 appears to be the disruption of long-range cooperativity, that is the fragmentation of collective wetting and drying transitions across the framework due to changes in intercage interactions (Figure SI27).

Furthermore, the disruption of the cooperative ZIF-8 network by bIm doping reveals two distinct regimes (Figure 5a–c and Figures SI20–SI25). At low B-type cage content (0–5%), even minimal substitution sharply reduces hysteresis (Figure 5a), signaling a breakdown of the network cooperativity regardless of the specific parameters chosen (Figures SI27–SI30). Beyond ∼10–15% B-type cages, the effect plateaus, indicating that a critical connectivity threshold has been crossed and the network becomes fundamentally fragmented (Figure SI26a). This loss of cooperativity coincides with a monotonic increase in both *P*_int_ and *P*_ext_ (Figure 5c) and a linear decrease in hysteresis (Figures 5a and SI30), which almost completely disappears for ca. 19%. This is reminiscent of a gradual change from a first-order to a second-order phase transition. These trends highlight the dominant role of A–A coupling in maintaining network stability.

We further investigated whether the disruptive effect of bIm linkers on network cooperativity could override other structural factors, such as crystallite size. For this, we simulated different network sizes at a fixed 5% B-type cage content. The results reveal that crystallite size dictates the transition pressures and hysteresis (Figure SI31), consistent with the explanation by Johnson et al.^34^ for pure ZIF-8, which involves decreasing surface-to-volume ratios. Additionally, we examined the homogeneity of B-type cages by testing both their spatial distribution within the crystallite and the statistical distribution of bIm linkers across B-type cages (i.e., increasing cage hydrophobicity while proportionally reducing the number of affected cages). The stochastic model simulations show that only a spatially uniform distribution of B-type cages reproduces the experimentally observed single-step intrusion–extrusion behavior and hysteresis trends (Figure SI32), whereas the homogeneous distribution of bIm across cages represents the most conservative scenario from the perspective of collective wetting behavior, i.e., the lowest possible bIm concentration required to obtain the experimentally measured changes in intrusion and extrusion behavior (Figure SI33).

All in all, our analysis, based on ab initio calculations, suggests some nonuniform distribution of linkers but the stochastic model has shown that this does not change the conclusions of our analysis and in fact this reinforces the argument on collective effect of linker doping on the wettability of ZIF-7-8 MOF.

Altogether, these results illustrate how local perturbations of the network in terms of connectivity and wetting properties by the addition of bIm linkers modulate global behavior in a composition-dependent manner, with the largest effects for small bIm contents. The stochastic simulations demonstrate that fluid behavior within a given cage is strongly influenced by the composition and hydration states of neighboring cages, indicating that wetting transitions are governed by nonlocal interactions. The present findings can be framed within the pore-limiting-diameter (PLD) versus largest cavity diameter (LCD) classification commonly used in MOF science.^51^ In ZIF-7-8 water wetting is governed not by isolated cages (LCD), but by the properties of the 6MR apertures that connect neighboring cavities, which act as a PLD-like control elements for cooperative intrusion and extrusion.

## Conclusions

In this work, we combined high-pressure intrusion-extrusion experiments, molecular dynamics simulations and stochastic modeling to elucidate the wetting behavior in ZIF-7-8 micropores.

Experimental isotherms revealed a clear dependence on bIm concentrations, with significant variations in wetting and dewetting parameters even when less than one linker per cage was substituted.

Atomistic simulations provided further insight, showing distinct free energy profiles for ZIF-8, ZIF-7-8 and ZIF-8-like cages. At 25 MPa, ZIF-8 favored a stable state with 39 water molecules, whereas ZIF-7-8 cages are in a metastable state accommodating only 29 molecules. In ZIF-8-like cages, the wet state persisted but with reduced stability, as evidenced by a 3-fold decrease in the free energy minimum (−19.8 *k*_B_ *T* to −6.1 *k*_B_ *T*).

Stochastic modeling demonstrated that in pure ZIF-8, strong intercage hydrogen bonding drives sharp, collective wetting-drying events. In contrast, the introduction of bIm weakens intercage interactions, suppressing cooperativity and leading to gradual, uncorrelated transitions. Remarkably, even minimal linker substitution was sufficient to disrupt the cooperative ZIF-8 network and sharply reduce hysteresis.

Altogether, these findings reveal the extraordinary sensitivity of cooperative wetting phenomena to subtle structural modifications, providing key insights for the rational design of nanoporous frameworks for energy storage, separation, catalysis, and related applications.

## Methods/Experimental

### Experimental Section

ZIF-7-8 synthesis at different bIm concentrations was achieved by modifying existing methods reported in the literature.^44,52^ The synthesis process was systematically optimized by adjusting reagent quantities and reaction times at a mild temperature of 110 °C.

Water intrusion/extrusion experiments were conducted using an AutoPore IV 9500 porosimeter (Micromeritics Instrument Corporation, Norcross, USA) to obtain PV isotherms (section SI2).

The linker concentration was determined via ^1^H Nuclear Magnetic Resonance (NMR). Approximately 2 mg of each sample was dissolved in *d*_4_-acetic acid before analysis.

Structural characterization was performed using X-ray diffraction (XRD) with a BRUKER-D8 DISCOVER diffractometer equipped with CuKα radiation (λ = 0.015406 nm). XRD patterns were recorded in 2θ steps over the range of 5–80° on powdered samples.

N_2_ adsorption–desorption isotherms were carried out on a micrometrics ASAP 2460 surface analyzer. For more information, see Section SI5 of the Supporting Information.

A Thermo Fisher Quanta 200 FEG Scanning Electron Microscope (SEM) was used to observe the crystallites of ZIF samples.

More detailed information regarding the synthesis procedure, as well as the porosimetry, NMR protocols, gas adsorption techinique, and SEM setup can be found in the Supporting Information Sections SI1–5 and SI9 and Figure SI34.

### Classical Molecular Dynamics

Classical MD simulations were performed using LAMMPS code.^53^ The computational slab consists of a supercell 2 × 2 × 4 of a unit cell. In the center of the slab, one mIm linker was replaced by one bIm to create the hybrid ZIF-7-8 with a very low concentration of bIm. The two external surfaces are saturated with hydrogen atoms. Two slabs of bulk water (5200 water molecules in total) are in contact with the ZIF-8 slab. The interatomic potential used is the one proposed by Zheng et al.^54^ in combination with the TIP4P/2005 model of water. The simulations were performed within the constant number of particles, pressure, and temperature ensemble (NPT). The pressure along the *x*–*y* plane of the slab is regulated by the Martyna-Tobias-Klein algorithm.^55^ Above and below the water slabs, two pistons apply hydrostatic pressure in orthogonal direction to the ZIF-8 slab; that implementation is required to avoid some artifacts related to conventional barostats in the presence of nonhomogeneous systems.^56^ The temperature is controlled via Nosè-Hoover chains thermostat.^57^ The presented setup has been successfully used in several previous publications.^33,36^ Additional information regarding MD and DFT simulations are reported in Supporting Information Sections SI7–SI10 and Figure SI35.

### The Stochastic Model

The stochastic model incorporated the capillary theory of wetting/drying in hydrophobic pores^58^ and the hydrophobic behavior of 6MR hydrogen-bonded apertures.^33^ Each cage was characterized by intrinsic wetting and drying barriers (ω_w_ and ω_d_), volume contributions (*v*_w_, *v*_d_), and interaction coefficients (*I*_AA_, *I*_AB_, *I*_BA_, *I*_BB_), which together determine its effective energy barrier under applied pressure and in the presence of neighboring wet cages.

The transition kinetics between dry and wet states were described using an Arrhenius-type rate

1 $$r_{w/d}=r_{0_{w/d}}e^{\Omega_{w/d}/k_{B}T}$$

although the classical Arrhenius formulation includes the Boltzmann constant *k*_B_ and temperature *T*, the present model expresses transition rates directly rather than characteristic times, effectively absorbing temperature effects into the energy barriers Ω and removing the explicit dependence on *k*_B_ *T*. Hence, the effective intrusion (wetting) and extrusion (drying) rates, *r*_w_ and *r*_d_, followed the expression $r_{w/d}=\frac{1}{t_{0}}e^{-\Omega }$ , where *t*_0_ denotes the characteristic attempt frequency. The term Ω accounts for cage-specific energy barriers, pressure-dependent contributions, and the hydration state of neighboring cages, thereby incorporating the hydrogen bonding interactions across the 6MR apertures connecting adjacent framework cages (further details in the Supporting Information).

The evolution of cage states, defined as dry (0) or wet (1), was governed by stochastic transitions driven by the calculated intrusion and extrusion rates. To simulate this behavior, transition probabilities were defined for each discrete time step d*t* as

2 $$p_{w}=1-e^{-dt/t_{w}}$$

3 $$p_{d}=1-e^{-dt/t_{d}}$$

where *p*_w_ and *p*_d_ represent the probabilities of wetting (filling) and drying (emptying), respectively. The characteristic times *t*_ω_ and *t*_d_ correspond to the inverse of the rate expressions *r*_w_ and *r*_d_, *t*_w_ = ^1^/*r*_w_ and *t*_d_ = 1/*r*_d_, ensuring consistency with energy-barrier-driven kinetics.

## Supplementary Material



## Notes

Details on ZIF-7-8 synthesis protocol, porosimetry technique, NMR protocol, XRD analysis, N_2_ adsoprtion-desorption technique and analysis, calculation of different bIm concentrations affecting SOD ZIF-7-8 cages, free energy calculation via restrained molecular dynamics, stochastic model, particle size analysis, theoretical analysis of bIm linkers distribution in a ZIF-7-8 crystallite (PDF)
