# Supplementary material for: Tuning the Wettability of Hydrophobic Metal–Organic Frameworks by Linker-Doping
Source: ACS Nano. 2026 May 4;20(19):14018–28. doi: 10.1021/acsnano.5c18554 (PMC13309003; doi:10.1021/acsnano.5c18554)
Supplement: Supplementary file 1 [file nn5c18554_si_001.pdf]

Supporting Information for:

Tuning the Wettability of Hydrophobic Metal-Organic Frameworks by Linker-Doping

Davide Caporale [a,b,†], Daniel Moreno-Rodríguez [c,†], Andrea Le Donne [d,†], Eder Amayuelas [a], Luis Bartolomé [a], Cléophée Gourmand [a], Gabriel Alejandro López [b], Kerman Gómez [a], Juan Miguel López del Amo [a], Antonio Tinti [e], Sebastiano Merchiori [d], Alberto Giacomello [c], Simone Meloni [d], and Yaroslav Grosu [a,f]

***a** Centre for Cooperative Research on Alternative Energies (CIC energiGUNE), Basque Research and Technology Alliance (BRTA), Alava Technology Park, Albert Einstein 48, 01510 Vitoria-Gasteiz, Spain*

***b** University of the Basque Country (UPV/EHU), Department of Physics, Bilbao, 48490, Spain.*

***c** Sapienza Università di Roma, Dipartimento di Ingegneria Meccanica e Aerospaziale, Rome, 00184, Italy.*

***d** Università degli Studi di Ferrara, Dipartimento di Scienze Chimiche, Farmaceutiche e Agrarie, Ferrara, Italy.*

***e** Laboratory of Molecular Simulation (LSMO), Institut des Sciences et Ingénierie Chimiques and Ecole Polytechnique Fédérale de Lausanne (EPFL), Rue de l'Industrie 17, 1951, Sion, Switzerland.*

***f** Institute of Chemistry, University of Silesia, Katowice, 40-006, Poland*

†These authors contributed equally to this work.

Corresponding Authors: Alberto Giacomello (alberto.giacomello@uniroma1.it), Simone Meloni (simone.meloni@unife.it), Yaroslav Grosu (ygrosu@cicenergigune.com

Contents

SI1 ZIF-7-8 Synthesis protocol 2

SI2 Porosimetry 2

SI3 NMR Protocol 3

SI4 X-ray diffraction analysis 4

SI5 N_2 adsorption-desorption isotherms 10

SI6 Calculations of different bIm concentrations affecting SOD ZIF-7-8 cages. 13

SI7 Free Energy Calculation via Restrained Molecular Dynamics 15

SI8 Stochastic Model 18

SI9. Particle size distribution analysis 37

SI10. Theoretical analysis of bIm linkers distribution in a ZIF-7-8 crystallite 38

Bibliography 43

SI1 ZIF-7-8 Synthesis protocol

ZIF-7-8 at different benzimidazole (bIm) concentrations was achieved by modifying existing method reported in the literature [1], [2]. The synthesis was conducted via a solvothermal method, systematically adjusting reagent quantities and reaction time at a mild temperature (110°C). Table SI1 reports the reagent quantities and the reaction time used for each sample. First, a solution was prepared by dissolving HCOONa, 2-methylimidazole (mIm), bIm in Methanol (MeOH). Separately, a second solution was prepared by dissolving $Zn(NO_3)2{\cdot}6H_2O$ in N,N-Dimethylformamide (DMF). The second solution was then slowly poured into the first and stirred for 10 minutes at room temperature. The resulting mixture was transferred to a 45 ml Teflon-lined autoclave and allowed to react at 110°C. After the reaction, the product was washed three times with MeOH (1 h per cycle). Finally, the samples were dried in an oven at 60 °C for 24 hours.

**NMR verified bIm content**	**HCOONa**	**mIm**	**bIm**	**(bIm/mIm) *100**	**MeOH**	**$Zn(NO_3)_2{\cdot}6H_2O$**	**DMF**	**Reaction time@ 110 °C**
1%	0.546 g	0.653 g	0.009 g	1.4%	20 mL	0.597 g	20 mL	20 h
1.5%	0.546 g	0.653 g	0.009 g	1.4%	20 mL	0.597 g	20 mL	20 h
2%	0.546 g	0.653 g	0.009 g	1.4%	20 mL	0.597 g	20 mL	2 h
9%	0.546 g	0.653 g	0.049 g	7.5%	20 mL	0.597 g	20 mL	2 h
13%	0.113 g	0.383 g	0.045 g	11.7%	20 mL	0.0991 g	20 mL	2 h
19%	0.111 g	0.372 g	0.053 g	14.2%	20 mL	1 g	20 mL	2 h
27%	0.111 g	0.35 g	0.067 g	19.1%	20 mL	1 g	20 mL	2 h
29%	0.111 g	0.3 g	0.084 g	28%	20 mL	1 g	20 mL	2 h

Table SI1. Reagent amounts, reagent ratios, and reaction times used for each sample in the synthesis.

SI2 Porosimetry

Porosimetry experiments were conducted to determine the intrusion-extrusion isotherms of water within ZIF-7-8. An AutoPore IV 9500 porosimeter (Micromeritics Instrument Corporation, Norcross, USA) was used for these measurements. Before testing, approximately 50 mg of each sample was enclosed in a flexible polyethylene capsule along with water. The capsules were placed in a penetrometer, where the pressure was initially reduced to 7 Pa and then filled with mercury. During high-pressure analysis, the pressure increased to 50 MPa. To ensure material stability,

three high-pressure cycles were performed. After testing, the powders were dried at 60 °C for 24 hours.

SI3 NMR Protocol

Solution NMR experiments were performed in deuterated acetic acid (CD_3COOD) at 300 K on a Bruker AVANCE III HD 300 MHz spectrometer equipped with a Bruker BBFO z-gradient 5 mm probe head. Proton spectra were acquired using the following parameters: 256 transients, a recycle delay of 10.0 s, an acquisition time of 5.45 s, and 64k data points.

NMR is a widely used technique for the quantitative analysis of molecular mixtures, as it provides direct information on relative concentrations. However, accurate quantification requires full relaxation of all NMR signals between scans to avoid errors caused by differential longitudinal relaxation (T_1). To ensure complete relaxation, the repetition delay (D_1) was set to at least 5 x T_1. T_1 values were determined via inversion recovery experiments before data acquisition.

For quantitative integration, isolated resonances with minimal spectral overlap were selected: the peak at 2.68 ppm was assigned to the methyl protons of mIm, and the peak at 7.86 ppm to two aromatic protons of bIm. Since the NMR integrals are proportional to the number of nuclei contributing, raw integrals do not directly reflect molar ratios. Each integral was normalised by dividing it by the number of protons associated with the corresponding signal. This normalisation ensures that calculated values represent the true molecular ratio rather than absolute signal intensity.

The total molar fraction was obtained by adding the normalised integrals. The molar percentage of each species was then calculated by dividing its normalised value by the total and multiplying by 100. This correction guarantees that the final percentages accurately reflect the actual mIm/bIm composition in solution. To ensure reproducibility, all experiments were conducted in triplicate, minimising experimental variability and enhancing statistical robustness.

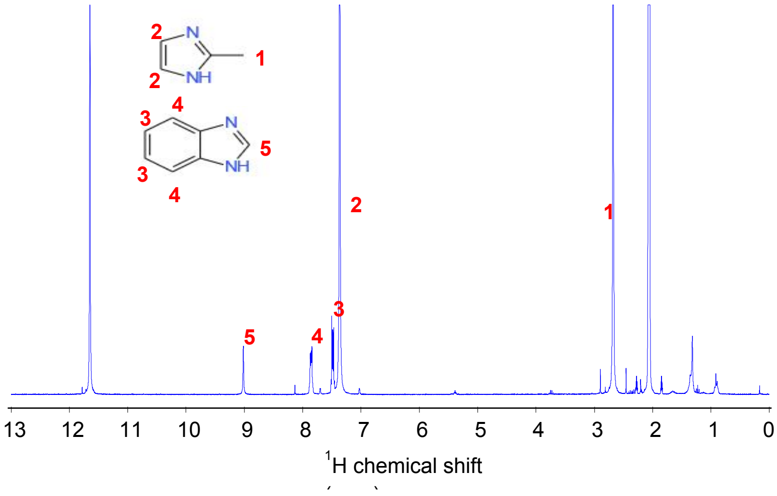

Figure SI1. ^{1}H experiment of the mixture containing bIm and mIm. The proton signals are labelled with numbers corresponding to the assigned resonances of each molecule.

The bIm/mIm ratios determined by ^{1}H NMR were found to correlate the nominal linker compositions targeted through the precursor ratios reported in Table SI1. However, non-negligible differences are evident, suggesting that nominal concentrations used in the synthesis must be verified by precise techniques such as NMR.

SI4 X-ray diffraction analysis

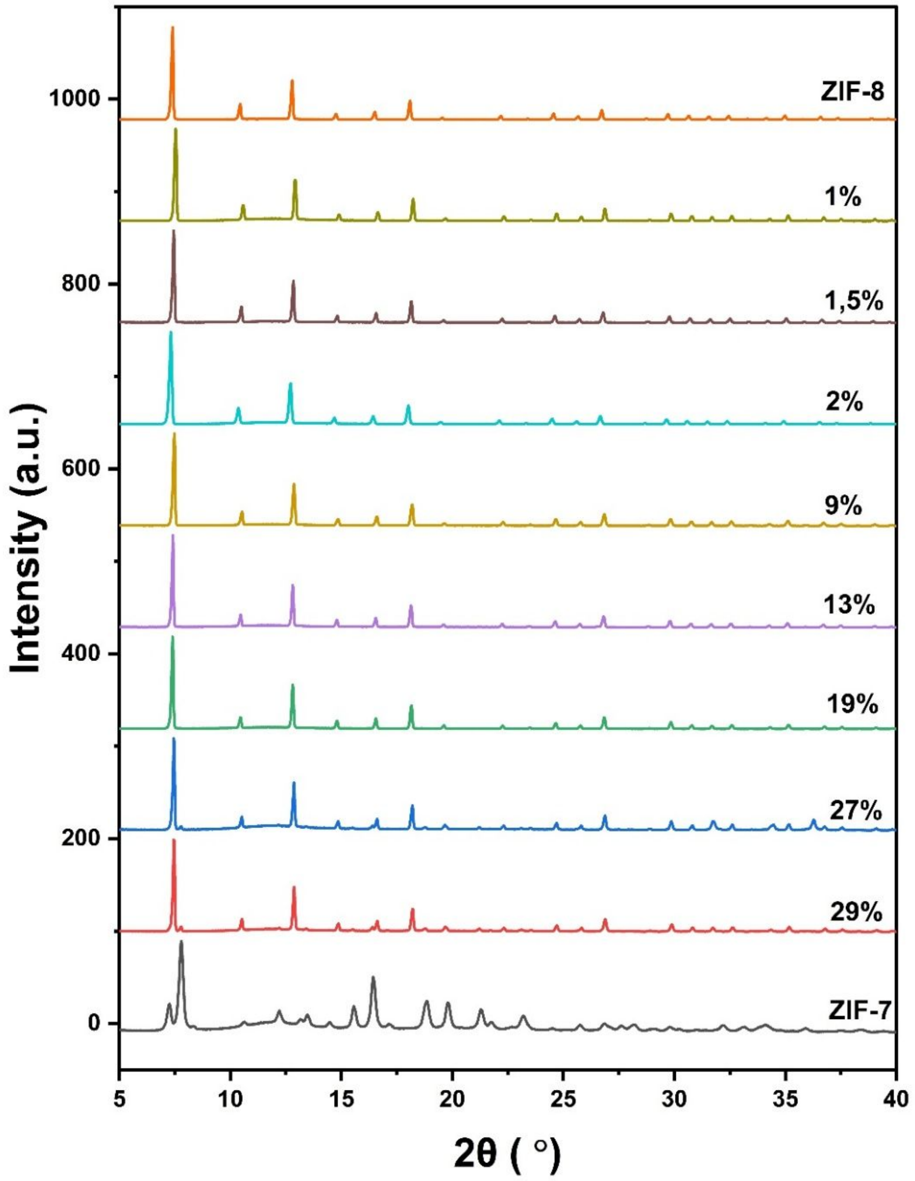

Figure SI2. XRD patterns of ZIF-7-8 hybrids.

Fig. SI2 shows the XRD patterns of the ZIF-7-8 hybrids. All recorded patterns confirm that the SOD topology is preserved across different hybridisations, with all samples exhibiting the same I4m space group. To further support this, Le Bail

refinements were performed, as shown in Figs. SI3 to SI11. Extra peaks are observed at 73% and 71% mIm concentrations, respectively. Comparing the 2θ positions of these peaks with those of the pure ZIF-7 pattern suggests the emergence of a secondary phase with a rhombohedral R3 space group, typical of ZIF-7. A notable observation is the shift in the peak position, which correlates with changes in the lattice parameter of the cubic cell (see Fig. SI12).

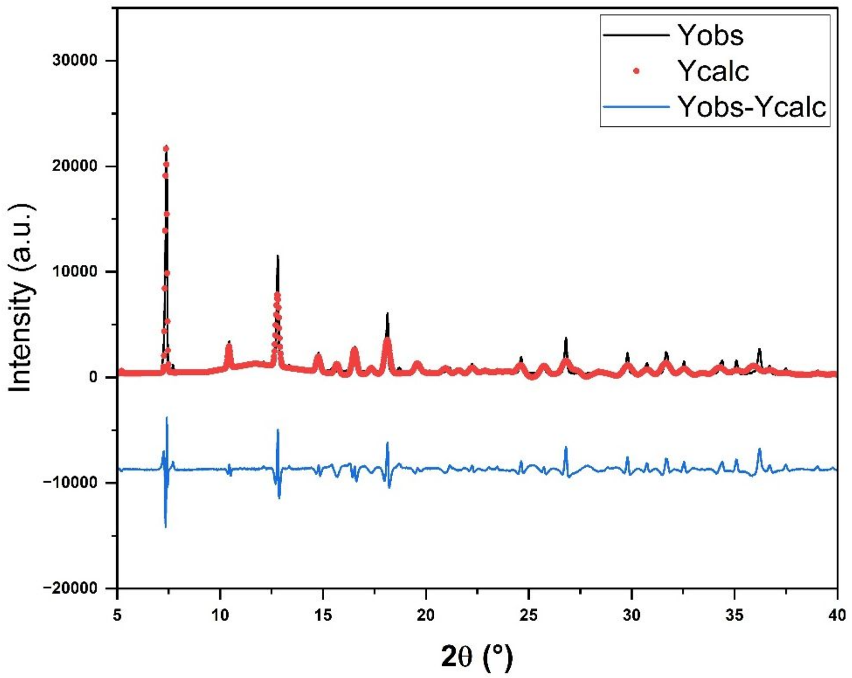

Figure SI3. Le Bail Refinement of the ZIF-7-8 sample with 29% bIm concentration.

Figure SI4. Le Bail Refinement of the ZIF-7-8 sample with 27% bIm concentration.

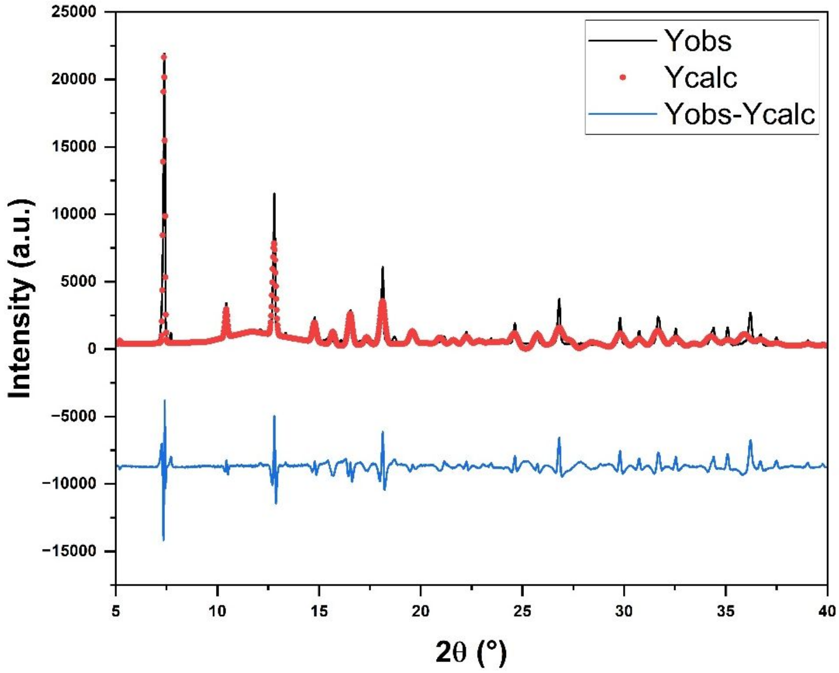

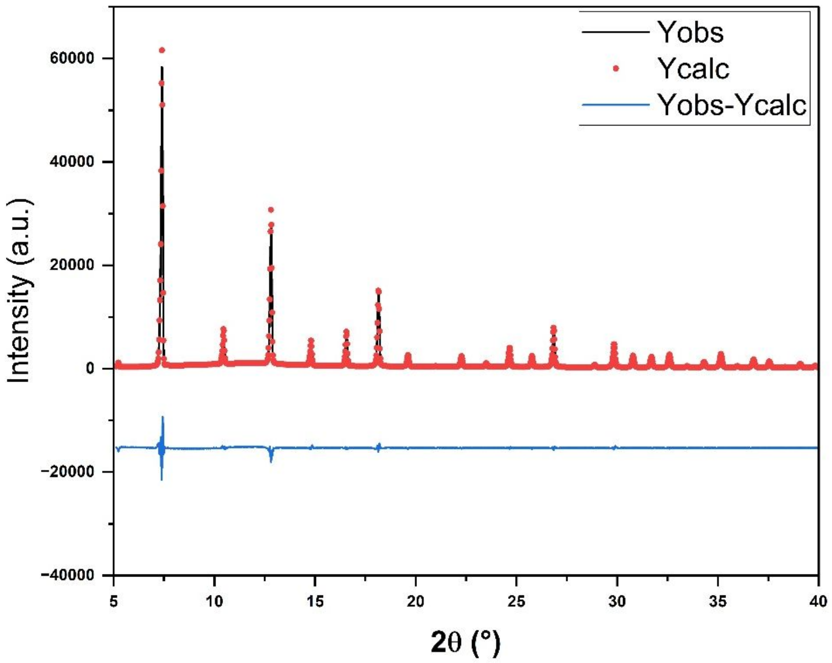

Figure SI5. Le Bail Refinement of the ZIF-7-8 sample with 19% bIm concentration.

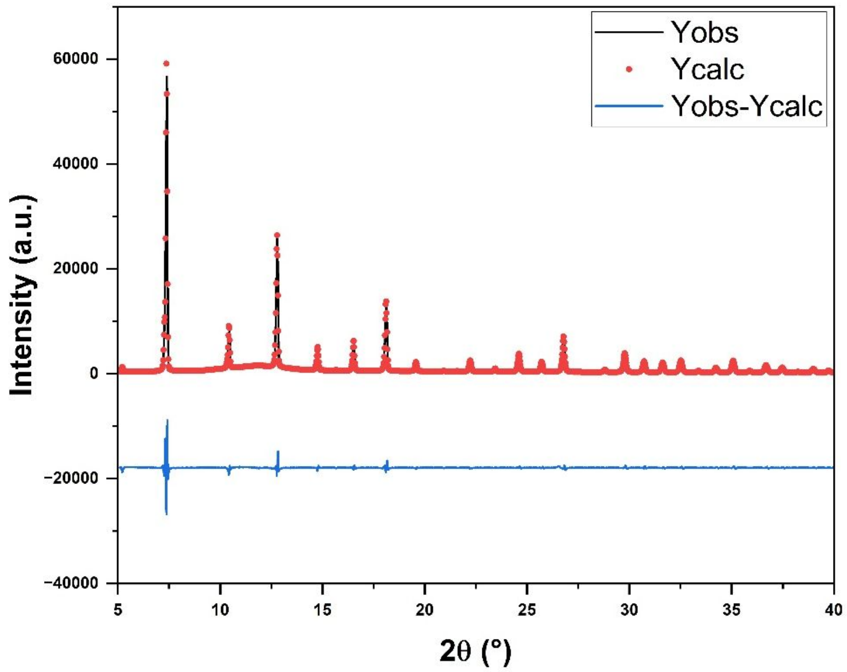

Figure SI6. Le Bail Refinement of the ZIF-7-8 sample with 13% bIm concentration.

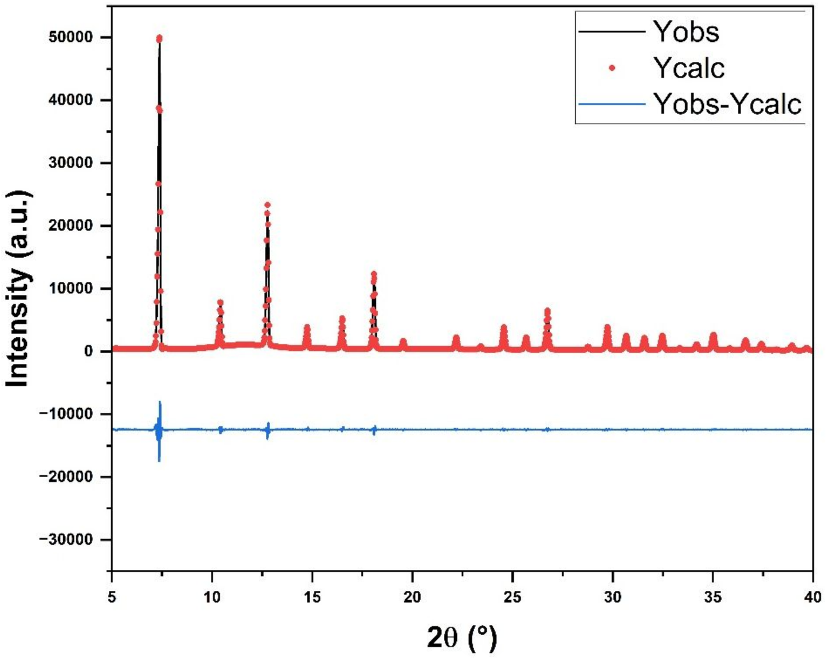

Figure SI7. Le Bail Refinement of the ZIF-7-8 sample with 9% bIm concentration.

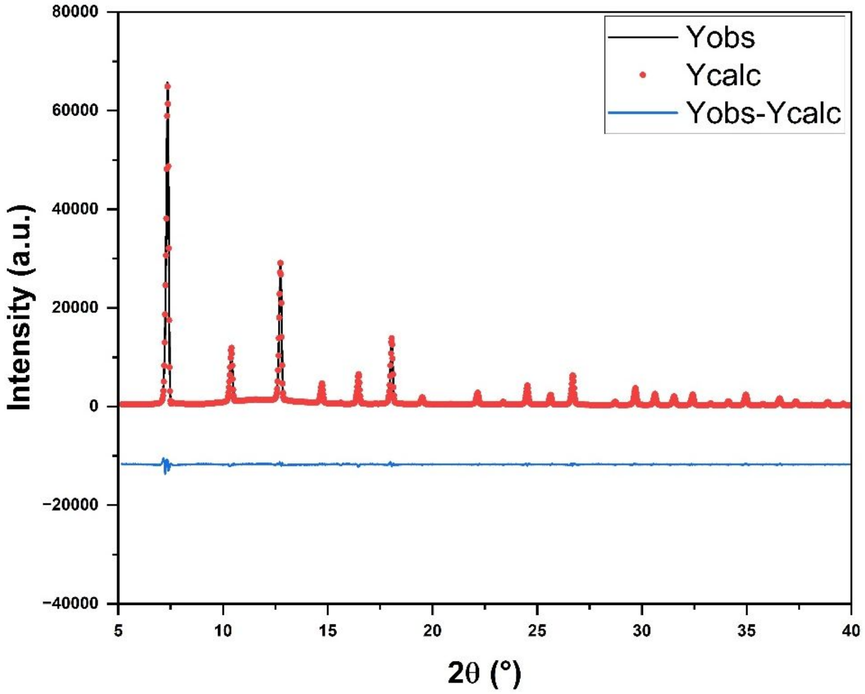

Figure SI8. Le Bail Refinement of the ZIF-7-8 with 2% bIm concentration.

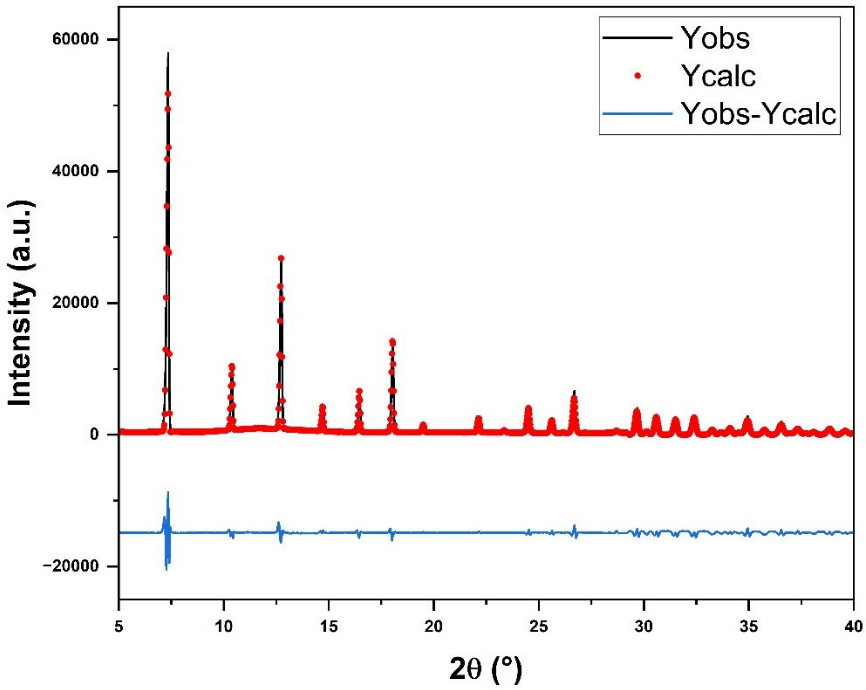

Figure SI9. Le Bail Refinement of ZIF-7-8 with 1,5% bIm concentration.

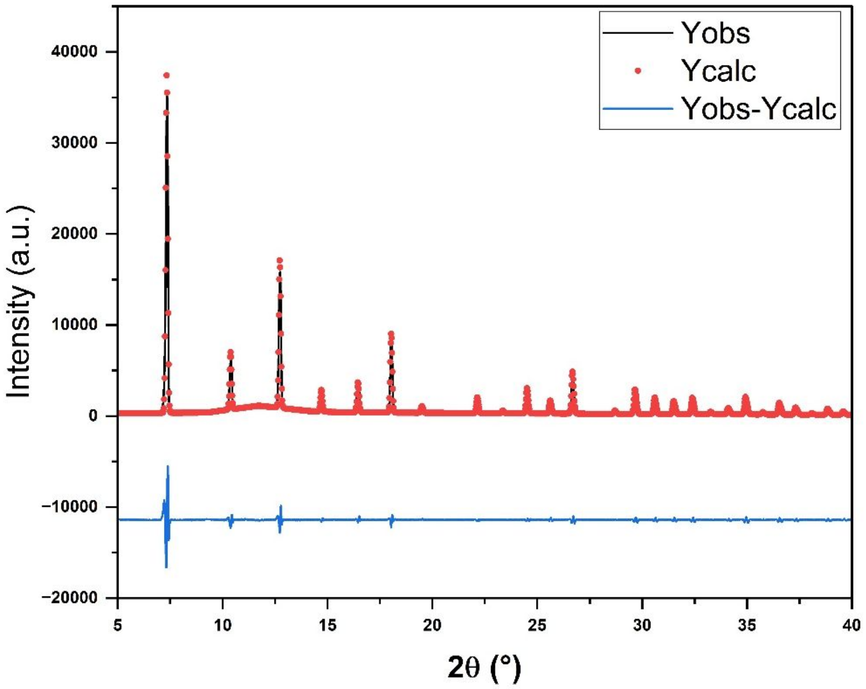

Figure SI10. Le Bail Refinement of ZIF-7-8 with 1% bIm concentration.

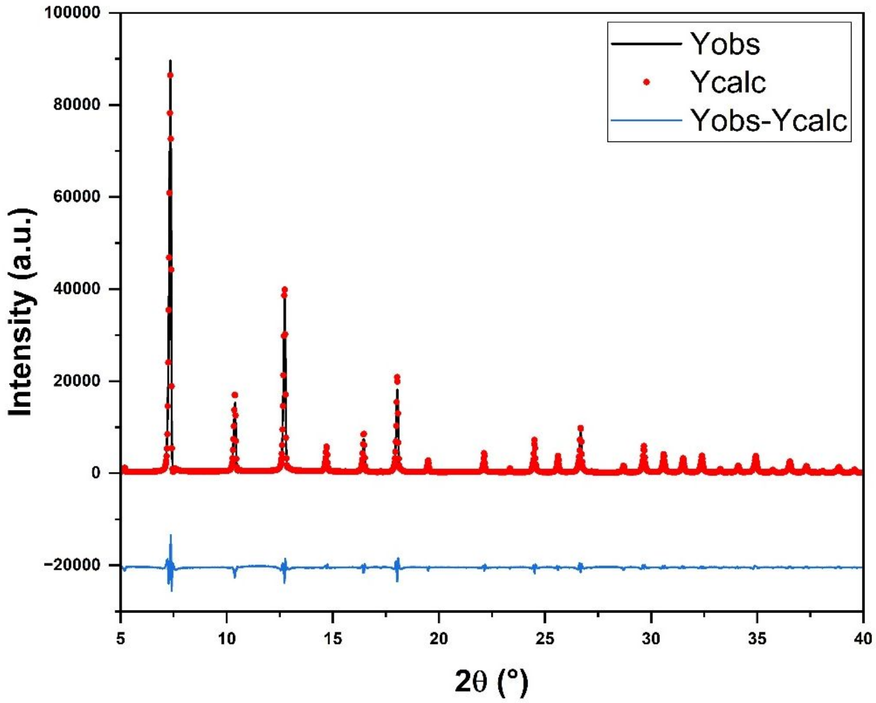

Figure SI11. Le Bail Refinement of ZIF-8.

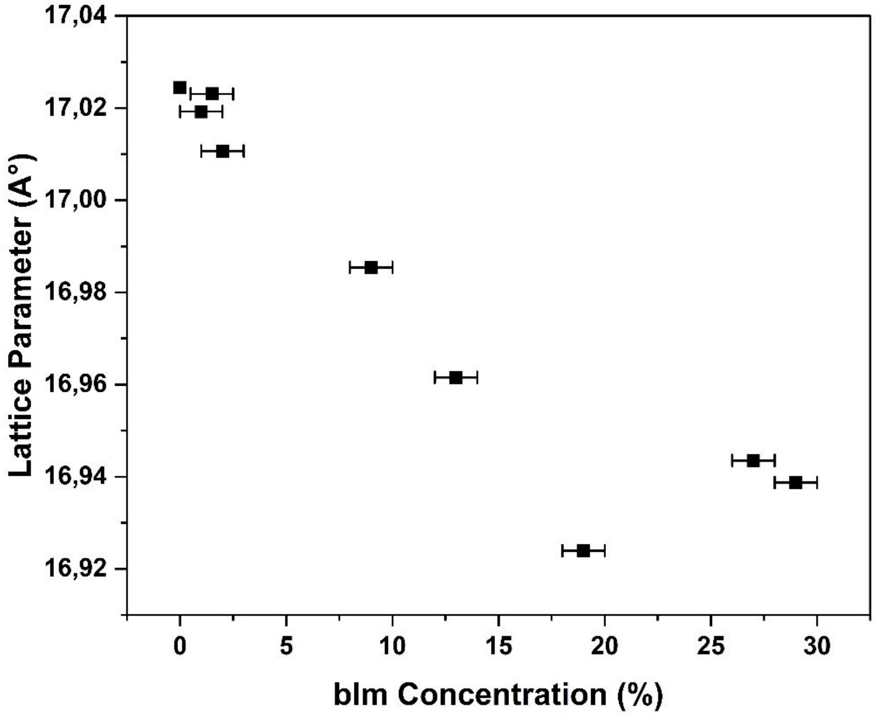

Figure SI12. Lattice Parameters of the ZIF-7-8 samples.

SI5 N_2 adsorption-desorption isotherms

Adsorption and desorption of N_2 at 77K was carried out to characterize the porous properties of the samples. Analysis was carried out on a Micromeritics ASAP 2460 surface analyzer. Prior to analysis, the samples were degassed for 24 hours at 100°C under vacuum. The specific surface area was determined using the Brunauer–Emmett–Teller (BET) equation in the P/P_0 = 0.05 – 0.20 relative pressure range. Pore volume was calculated at P/P_0 = 0.99 and micropore volume was determined using t-plot method. Pore size distributions were calculated using classical density functional theory (CDFT) [3], [4] for which a qualitative comparison of the samples will be provided. The DFT used here must not be confused with the DFT model used later to simulate at the electronic level ZIF-7-8 structure.

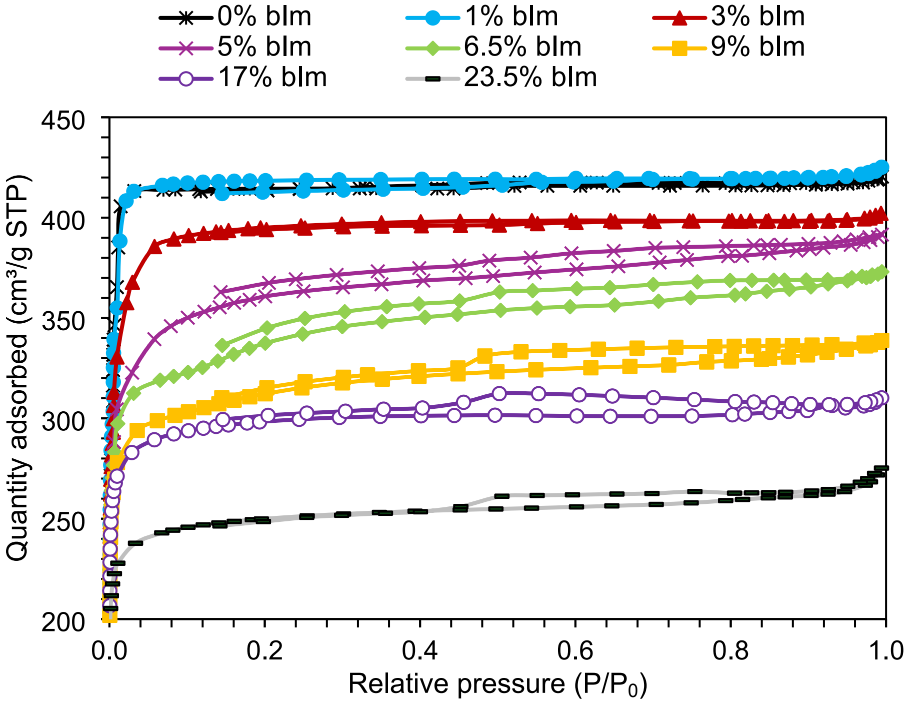

Figure SI13. N_2 adsorption-desorption isotherms of ZIF-7-8 samples with different bIm concentrations.

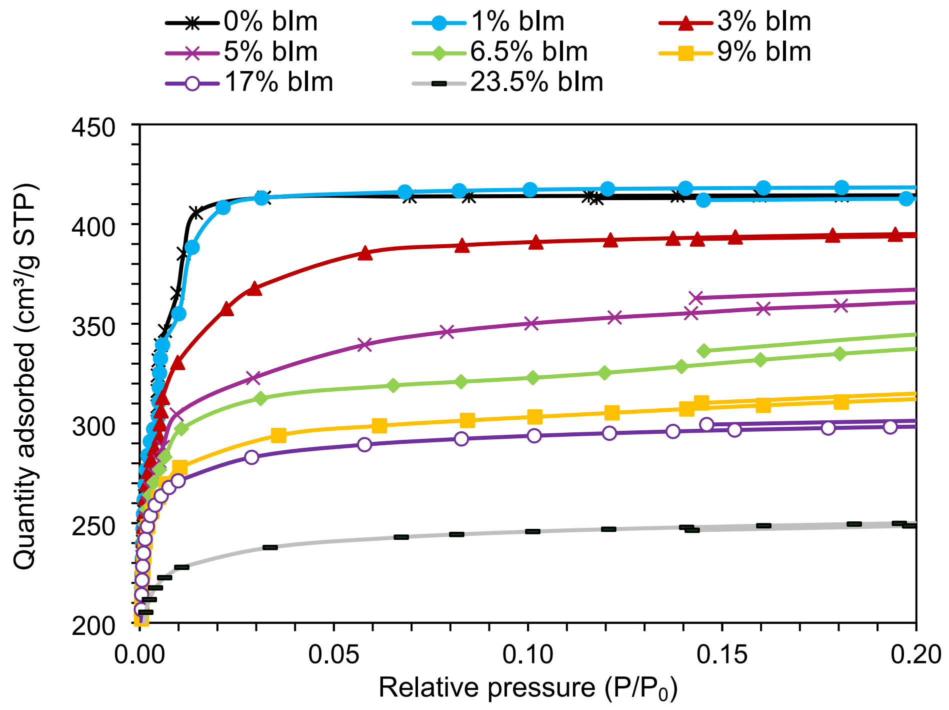

Figure SI14. Zoomed view of the adsorption-desorption isotherms of ZIF-7-8 samples with different bIm concentrations.

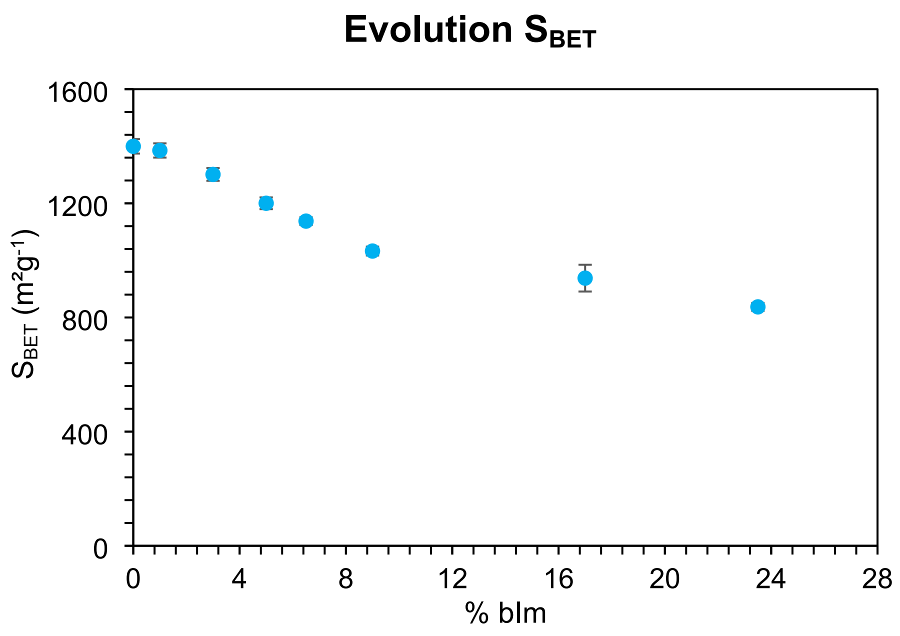

Figure SI15. Evolution of BET surface area (S_{BET}) of ZIF-7-8 samples with different bIm concentrations.

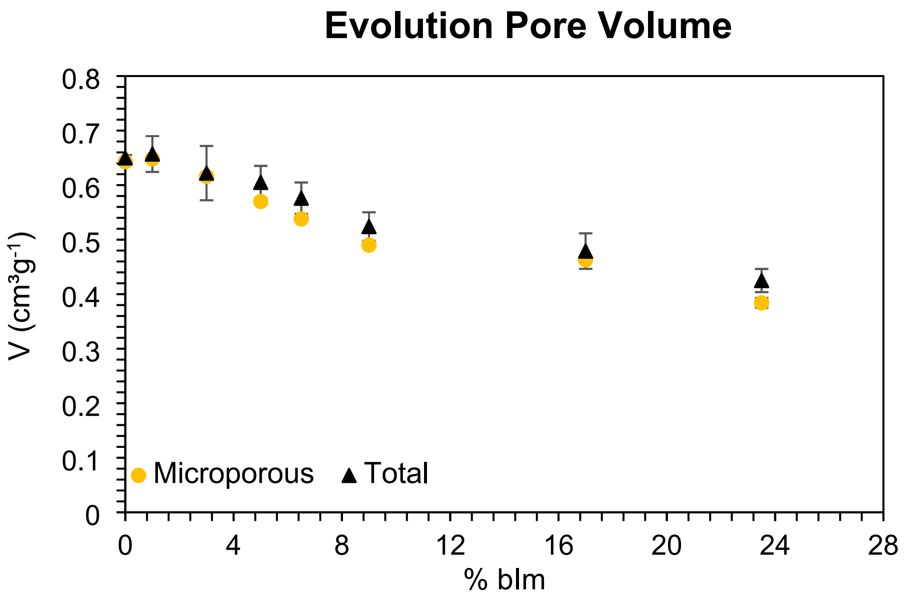

Figure SI16. Evolution of Pore Volume of ZIF-7-8 samples with different bIm concentrations.

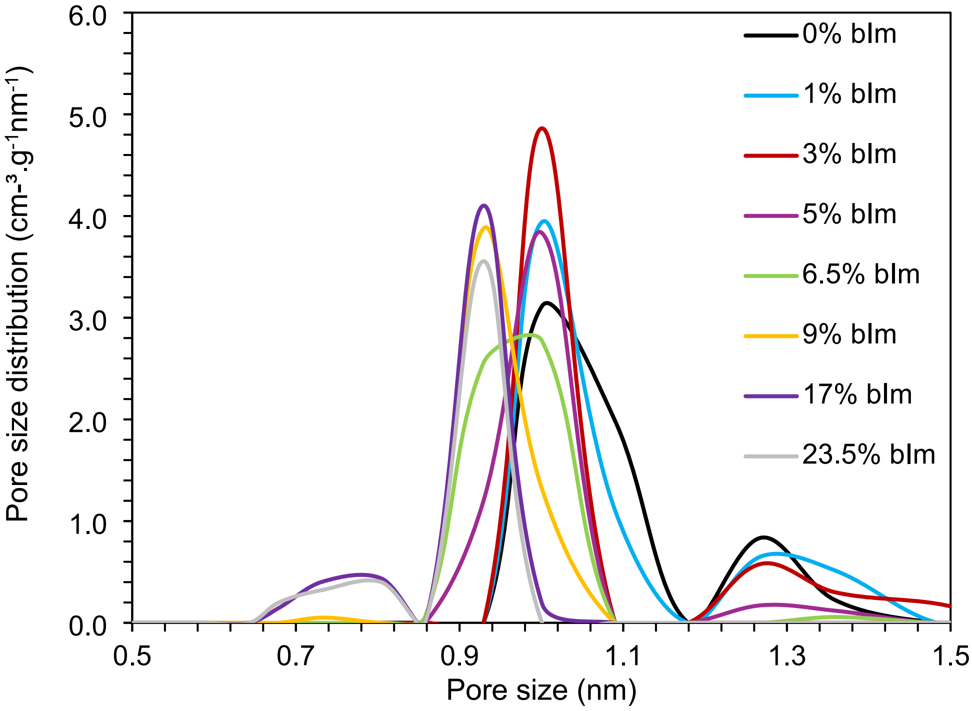

Figure SI17. Pore size distributions of ZIF-7-8 samples with different bIm concentrations.

As can be seen from Fig. SI17 pore size distribution remains the same for all the samples within ± 1Å. These Figure should be considered with caution, as 1)

absolute values of PSD obtained from gas adsorption is highly model dependent, but by applying the same model, one can reliably see that PSD has not changed for all the sample and 2) gate-opening effect result in additional peaks on PSD for some samples, that are not related to pore size, but to flexibility effects.

SI6 Calculations of different bIm concentrations affecting SOD ZIF-7-8 cages.

We quantify, under an ideal topological model, how many sodalite cages (SOD cages) are affected (i.e., share at least one modified organic linker) when a given fraction of mIm linkers is replaced by a bIm linker in ZIF-8. We adopted a deterministic, homogeneous distribution of bIm linkers designed to maximize the number of distinct cages touched at fixed linker count. This provides the best case (upper-bound) coverage curve that complements the random substitution expectation reported elsewhere [5].

Topological model and assumptions

1. Topology-only abstraction: ZIF-8 is abstracted to the SOD net (4-connected 3-periodic model). Nodes represent metal/SBU vertices; edges represent organic linkers. Geometry, flexibility, and chemistry beyond connectivity are ignored.
2. Counting identities for SOD:
    - Each cage is a truncated octahedron bounded by 36 edges (organic linkers).
    - Each edge is shared by three cages.
    - In a periodic $n \times n \times n$ block of SOD cages:

    $$C = n^3 (\text{number of cages}), M = 12C (\text{number of linkers}).$$

    The latter follows from “edges per cage ÷ sharing”: $M = (36C)/3 = 12C$.
3. Periodicity: Symmetry restrictions and periodicity is not considered in this approach as bIm is randomly distributed as previously reported. Every edge is shared by exactly three cages.
4. Definition of “affected cage”: A cage is affected if at least one of its 36 incident linkers is of the modified type.
5. Deterministic placement rule (edge-disjoint design): To maximize distinct cages affected by a fixed number m of bIm linkers, choose linkers edge-disjoint so that no two chosen bIm linkers touch the same cage. Since each linker touches three cages, the theoretical maximum of affected cages is

    $$N_{\text{aff}}(m) = \min(3m, C).$$

This maximum is achievable in periodic SOD by tiling a small periodic pattern (e.g., a 3×3×3 motif) so that selected edges are mutually disjoint and uniformly distributed.

In this work we use a 9x9x9 cages slab:

For $n = 9$,

$$C = 9^3 = 729, M = 12C = 8748\ cages.$$

Minimum concentration for 100% cage coverage:

To touch every cage at least once, we need at least one modified linker per 3 cages:

$m_{\min} = \lceil \frac{C}{3} \rceil = \lceil \frac{729}{3} \rceil = 243$, hence the critical concentration

$$p_{\text{crit}} = \frac{m_{\min}}{M} = \frac{243}{8748} = \frac{1}{36} \approx 2.777\%.$$

This value is size-independent for periodic SOD (it is a topological constant).

Coverage at different low bIm concentrations:

For each concentration target (p), take $m = \lfloor pM \rfloor$, place m edges edge-disjoint and evenly spaced, and compute

$$N_{\text{aff}}(m) = \min(3m,\ 729), \%\ \text{cages} = 100 \times \frac{N_{\text{aff}}(m)}{729}.$$

Table SI2.Statistical analysis of the percentage of cages influenced by the presence of bIm as a function of linker concentration.

**Target p**	$\boldsymbol{m = \lfloor pM \rfloor}$	**Achieved conc. $\boldsymbol{100\,m/M}$**	**Affected cages $\boldsymbol{N_{\text{aff}}(m)}$**	**%cages affected**
**0.5%**	43	0.4915%	129	17.695%
**1.0%**	87	0.9945%	261	35.802%
**1.5%**	131	1.4975%	393	53.909%
**2.0%**	174	1.9890%	522	71.605%
**2.5%**	218	2.4920%	654	89.712%
**3.0%**	262	2.9950%	729 (saturation)	100.000%

Notes:

- Until saturation, the affected-cage percentage simplifies to

$$\% \text{ cages} = 100 \times \frac{3m}{C} = 100 \times \frac{m}{243}.$$

- At and above $m = 243$(≈2.777%), coverage saturates at 100%; additional modified linkers do not increase the number of affected cages under the ≥1-linker definition.

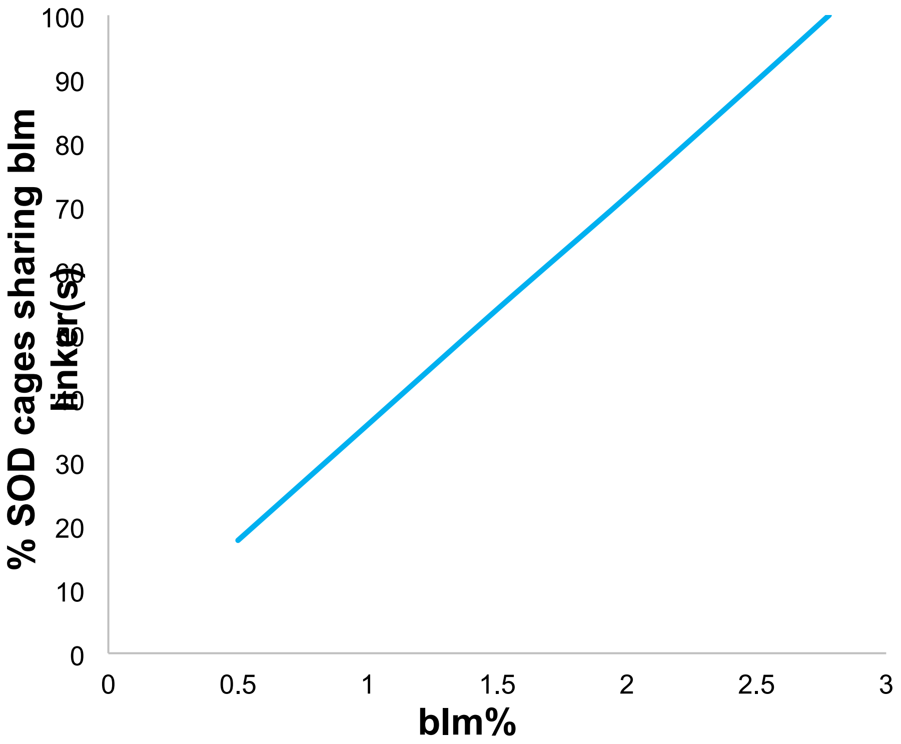

Figure SI18. Linear dependence of coverage of SOD cages (sharing at least one bIm linker) with concentration of bIm linkers.

Some limitations and intended use for this approach should be noted. First, the deterministic result is a best-case upper bound at fixed m. It overestimates coverage relative to random substitution (where multiple modified linkers often touch the same cage). The model is topological; geometric strain, linker orientation, and chemical preferences are neglected. Periodicity eliminates surface effects; for finite crystallites the edge-sharing relation is altered at the surface and must be handled separately. Despite these limitations, the deterministic curve is valuable as a design limit and for benchmarking simulations that enforce homogeneity constraints.

SI7 Free Energy Calculation via Restrained Molecular Dynamics

To compute the Landau free energy profile as a function of the number of water molecules inside a ZIF-7-8 cage, restrained molecular dynamics (RMD) simulations were employed. This method enables sampling across different filling levels by applying constraints that guide the system toward configurations that are otherwise

highly improbable within the timescales of conventional molecular dynamics (MD). In what follows, we present a step-by-step outline for reconstructing the free energy profile using RMD.

In a given ensemble, any thermodynamic potential can be related to the logarithm of a corresponding probability density function, *P*. In this study, we sample the probability of observing a target number of water molecules within a specific volume – namely, the accessible pore volume of a ZIF-7-8 cage. We define the probability density function for the target number of water molecules, $M(N_{\mathrm{H_2O}})$, as the likelihood that a ZIF-7-8 cage contains exactly $N_{\mathrm{H_2O}}$ water molecules. This can be expressed in terms of the ensemble distribution $m(\boldsymbol{r})$, which denotes the probability density of finding the system in a configuration specified by the 6N-dimensional atomic coordinate vector $\boldsymbol{r}$:

$$M(N^*_{\mathrm{H_2O}}) = \int m(\boldsymbol{r})\delta(\hat{N}_{\mathrm{H_2O}}(\boldsymbol{r}) - N^*_{\mathrm{H_2O}})\mathrm{d}\boldsymbol{r}$$

Wherein the Dirac delta function δ allows us to specifically choose configurations corresponding to our desired level of filling, with $\hat{N}_{H_2O}(\boldsymbol{r})$ counting the number of molecules in the cavity at the configuration $\boldsymbol{r}$. As mentioned above, we now associate the logarithm of probability density with the Landau free energy, G:

$$G(N^*_{\mathrm{H_2O}}) = -k_{\mathrm{B}}T\log M(N^*_{\mathrm{H_2O}})$$

Here, $k_{\mathrm{B}}T$ represents the thermal energy at temperature *T*, with k_{B} being the Boltzmann constant.

In principle, the free energy $G(N^*_{\mathrm{H_2O}})$ can be estimated from simulation data by computing the histogram associated with $M(N^*_{\mathrm{H_2O}})$, assuming that sufficient sampling is available to construct such a histogram. However, it is well known that this direct approach is computationally inefficient.

To circumvent this problem, here we employ restrained molecular dynamics (RMD), a technique that introduces an augmented potential into the system to enforce exploration of configurations associated with a specific target property, the number of water molecules within a particular cage, in this case. Over the course of the simulation trajectory, RMD enables the sampling of the conditional distribution $\mu(\boldsymbol{r}; N^*_{\mathrm{H_2O}})$, the probability density of being at the configuration $\boldsymbol{r}$ while the target cavity contains $N^*_{\mathrm{H_2O}}$ water molecules, which, in turn, allows us to compute any statistical property of

interest, including the free energy. Assuming the system follows a canonical ensemble, the probability distribution of a given microstate $\boldsymbol{r}$ can be expressed as:

$$\mu(\boldsymbol{r}) = \frac{\exp\left[-\frac{V(\boldsymbol{r})}{k_{\mathrm{B}}T}\right]}{\int \mathrm{d}\boldsymbol{r} \exp\left[-\frac{V(\boldsymbol{r})}{k_{\mathrm{B}}T}\right]}$$

where $V(\boldsymbol{r})$ is the potential energy of configuration $\boldsymbol{r}$, k_{B} is the Boltzmann constant, and T is the absolute temperature.

Herein, the potential energy function $V(\boldsymbol{r})$ is defined by the chosen force-field parameters which describe how the ZIF-7-8 framework and water molecules interact – both through internal bonding and external non-bonded intermolecular forces. Substituting this definition into the expression for $M\left(N^*{}_{H_2O}\right)$, we obtain:

$$M\left(N^*{}_{\mathrm{H_2O}}\right) = \frac{\int \mathrm{d}\boldsymbol{r}\ \exp\left[-\frac{V(\boldsymbol{r})}{k_{\mathrm{B}}T}\right] \delta\left(\hat{N}_{\mathrm{H_2O}}(\boldsymbol{r}) - N^*{}_{\mathrm{H_2O}}\right)}{\int \mathrm{d}\boldsymbol{r} \exp\left[-\frac{V(\boldsymbol{r})}{k_{\mathrm{B}}T}\right]}$$

Differentiating the Landau free energy (Eq.9) with respect to $N^*{}_{H_2O}$, and applying the chain rule, we obtain:

$$\frac{\mathrm{d}G(N^*{}_{\mathrm{H_2O}})}{\mathrm{d}N^*{}_{\mathrm{H_2O}}} = -\,k_{\mathrm{B}}T \frac{\int \mathrm{d}\boldsymbol{r} \exp\left[-\frac{V(\boldsymbol{r})}{k_B T}\right] \frac{\mathrm{d}}{\mathrm{d}N^*{}_{\mathrm{H_2O}}} \left[\delta\left(\hat{N}_{\mathrm{H_2O}}(\boldsymbol{r}) - N^*{}_{\mathrm{H_2O}}\right)\right]}{\int \mathrm{d}\boldsymbol{r} \exp\left[-\frac{V(\boldsymbol{r})}{k_{\mathrm{B}}T}\right]}$$

As shown below, this derivative $\frac{\mathrm{d}G(N^*{}_{\mathrm{H_2O}})}{\mathrm{d}N^*{}_{\mathrm{H_2O}}}$ can be estimated using RMD simulations and numerically integrated to recover the free energy $G(N^*{}_{\mathrm{H_2O}})$.

To make Eq.14 more amenable to numerical estimation, the Dirac delta function is approximated using a Gaussian distribution:

$$\delta\left(\hat{N}_{\mathrm{H_2O}}(\boldsymbol{r}) - N^*{}_{\mathrm{H_2O}}\right) \approx g_\lambda\left(\widehat{N_{\mathrm{H_2O}}} - N^*{}_{\mathrm{H_2O}}\right) = \sqrt{\frac{2\pi k_{\mathrm{B}}T}{\lambda}} exp\left[-\frac{\lambda}{2}\frac{\left(\hat{N}_{\mathrm{H_2O}}(\boldsymbol{r}) - N^*{}_{\mathrm{H_2O}}\right)^2}{k_{\mathrm{B}}T}\right]$$

where $k_{\mathrm{B}}T/\lambda$ is the variance of the Gaussian function.

Applying this approximation, the derivative of the free energy becomes:

$$\frac{\mathrm{d}G(N^*{}_{H_2O})}{\mathrm{d}N^*{}_{\mathrm{H_2O}}} = \frac{\int \mathrm{d}\boldsymbol{r}\ \frac{\lambda}{k_{\mathrm{B}}T}\left(\hat{N}_{\mathrm{H_2O}}(\boldsymbol{r}) - N^*{}_{\mathrm{H_2O}}\right) exp\left[-\frac{V(\boldsymbol{r}) + \frac{\lambda}{2}\left(\hat{N}_{\mathrm{H_2O}}(\boldsymbol{r}) - N^*{}_{\mathrm{H_2O}}\right)^2}{k_{\mathrm{B}}T}\right]}{\int \mathrm{d}\boldsymbol{r}\ exp\left[-\frac{V(\boldsymbol{r}) + \frac{\lambda}{2}\left(\hat{N}_{\mathrm{H_2O}}(\boldsymbol{r}) - N^*{}_{\mathrm{H_2O}}\right)^2}{k_{\mathrm{B}}T}\right]}$$

This expression shows that the derivative of the free energy can be computed as the ensemble average of the observable $\lambda/k_{\mathrm{B}}T\left(\hat{N}_{\mathrm{H_2O}}(\boldsymbol{r}) - N^*{}_{\mathrm{H_2O}}\right)$ under an augmented potential $V(\boldsymbol{r}) + \frac{\lambda}{2}\left(\hat{N}_{\mathrm{H_2O}}(\boldsymbol{r}) - N^*{}_{\mathrm{H_2O}}\right)^2$.

In practice, this derivative can be estimated at each desired value of water occupancy by averaging the observable $\lambda/k_{\mathrm{B}}T\left(\hat{N}_{\mathrm{H_2O}}(\boldsymbol{r}) - N^*{}_{\mathrm{H_2O}}\right)$ over an RMD trajectory. Finally, the free energy profile $G(N^*{}_{\mathrm{H_2O}})$ is obtained by numerically integrating the derivative using the trapezoidal rule.

SI8 Stochastic Model

The computational model implements a stochastic representation of the SOD framework characteristic of ZIF-8, where each structural cage (modelled as a node) connects to up to eight neighbouring cages through 6MR apertures.

The network is generated using a growth algorithm that begins with a seed node and expands outward in a stochastic manner (e.g., through 10 iterations, generating 2331 nodes). As new nodes are added, they are probabilistically assigned to a cage type, either type A or B, according to a predefined B-type fraction, which in this study ranged from 0 to 30%.

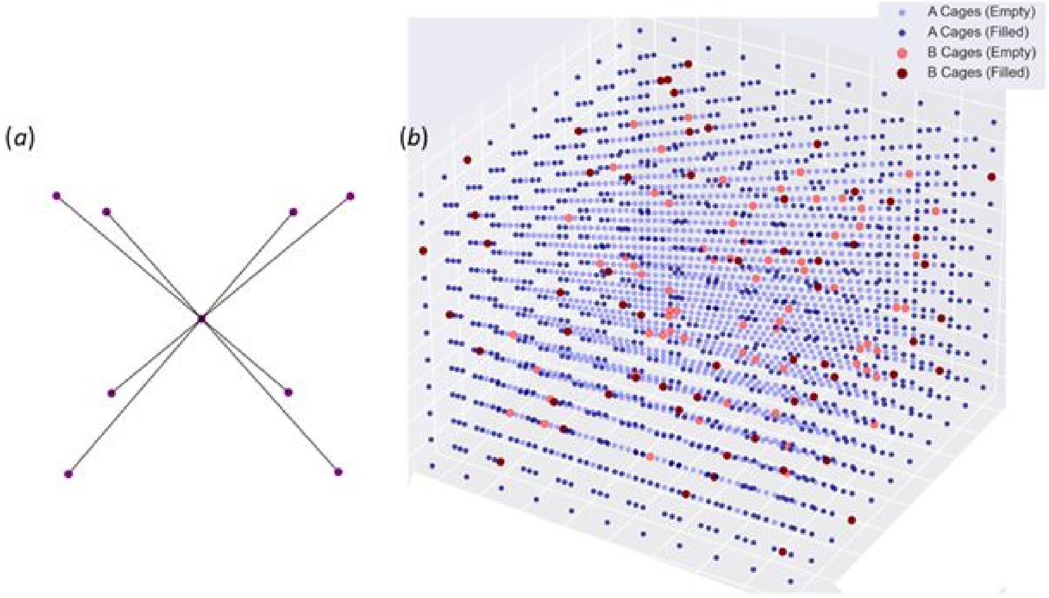

Figure SI19. (a) Schematic of the local ZIF-8 cage connectivity, where each cage (node) is linked up to eight nearest neighbours through 6MR apertures, forming the SOD topology. Three-dimensional representation of the ZIF-8 network used in the simulations (with 2331 nodes). Each point corresponds to a single cage, colour-coded by cage type and hydration state: empty A-type cages (light blue), filled A-type (dark blue), empty B-type (pink), and filled B-type (red).

Each cage type is characterised by a distinct set of parameters governing local transition behaviour under applied pressure. These include intrinsic energy barriers for wetting/drying ($\omega_{\mathrm{w}/\mathrm{d}}$), the volume changes associated with intrusion and extrusion (v_{w} and v_{d}), and neighbour interaction weights ($\mathrm{I_{AA}}, \mathrm{I_{AB}}, \mathrm{I_{BA}}, \text{and } \mathrm{I_{BB}}$), which quantify how the presence of wet neighbours of a given type affects the local cage's wetting probability.

Once the network is generated, the simulation proceeds through a time evolution loop governed by discrete time steps of length $\mathrm{d}t$. At each step, every node evaluates the probability of transitioning between dry and wet states. This is done by first computing an effective energy barrier, $\Omega_{w/d}$, which reflects the node's local environment.

$$\Omega_{\mathrm{w}} = \left(\omega_{\mathrm{w}} - P \cdot v_{\mathrm{w}} + \left(\frac{n_{\max}}{2} - n\right) \cdot I\right)$$

$$\Omega_{\mathrm{d}} = \left(\omega_{\mathrm{d}} + P \cdot v_{\mathrm{d}} + \left(n - \frac{n_{\max}}{2}\right) \cdot I\right)$$

The barrier depends on the cage type, the applied pressure, and the hydration state of neighbouring nodes, counted separately by type (wet A and wet B). Interaction energies between neighbours are defined through a parameter I, which captures the cooperative hydrogen bonding effects across the 6MR apertures:

$$I = \frac{n_{\mathrm{A}}(n_{\mathrm{A}}\mathrm{I}_{\mathrm{AA}} + n_{\mathrm{B}}\mathrm{I}_{\mathrm{AB}}) + n_{B}(n_{\mathrm{A}}\mathrm{I}_{\mathrm{BA}} + n_{\mathrm{B}}\mathrm{I}_{\mathrm{BB})}}{n^{2}{}_{\mathrm{max}}}$$

where $n_{\mathrm{A}}\ and\ n_{\mathrm{B}}$ denote the number of neighbouring type A and type B cages, respectively. The terms $\mathrm{I}_{\mathrm{AA},}\ \mathrm{I}_{\mathrm{AB}},\ \mathrm{I}_{\mathrm{BA}},$ and I_{BB} are the interaction coefficients between different cage types, and n_{max} is the maximum number of neighbouring cages, used for normalisation.

The resulting energy barrier is used to compute the transition rates r_{w} and r_{d}, where t_0 is the characteristic attempt frequency:

$$r_w = \frac{1}{t_0} e^{-\Omega_{\mathrm{w}}}$$

$$r_{\mathrm{d}} = \frac{1}{t_0} e^{-\Omega_{\mathrm{d}}}$$

From this, the transition probabilities over the time step are calculated via:

$$p_{\mathrm{w}} = 1 - e^{-\mathrm{d}t/t_w}$$

$$p_{\mathrm{d}} = 1 - e^{-\mathrm{d}t/t_{\mathrm{d}}}$$

Following the computation of transition probabilities, each node independently determines whether a state change occurs. Dry nodes may become wet (representing intrusion), while wet nodes may return to the dry state (extrusion), according to the probabilistic rules derived from Arrhenius kinetics. This stochastic update process is repeated across the entire network, allowing local cooperative phenomena to emerge organically without the need for global synchronisation. Hence, the model performs a continuous, linear pressure scan from 0 MPa to 50 MPa and back, spanning 120.000 time-steps and a duration of roughly tens of nanoseconds to a few microseconds (given a time step of 0.4 times a characteristic timescale regarding wetting of hydrophobic nanopores of about $10^{-10}\ to\ 10^{-12}$ s [6]).

Some insights from the stochastic model results:

Figs. SI20 to SI25 present simulated *PV*-isotherms for ZIF-7-8 frameworks with varying B concentrations from 0-30% using a stochastic model. Three key trends are observed in the figures: (a) the hysteresis decreases even at low B%; (b) intrusion-extrusion curves show higher intrusion and extrusion pressures with increasing B%; (c) normalised filling curves demonstrate loop narrowing, indicating diminished energy dissipation. The loop shrinkage reflects disrupted inter-cage interaction due to B substitution, leading to lower cooperativity and diminished hysteresis energy; (d) P_{int}

$and\ P_{\text{ext}}$ generally increase with B%. The P_{ext} slope rate is slower than P_{int} and varies with the parameters (e.g., $\omega_{\text{w}/\text{d}}$ and $I_{\text{AA}/\text{BB}}$).

The dry bubble tracking analysis, Fig. SI26, provides a microscopic view of how network cooperativity evolves with the B% content. At low B% concentrations (0-5%), the system exhibits strong A-A inter-cage coupling, enabling the formation of large, well-connected dry clusters that undergo sharp, collective collapse at the intrusion transition. This is reflected by a low number of large bubbles (Fig. SI26a), large average and maximum bubble sizes (Fig. SI26b-c), and high internal connectivity (Fig. SI26d). The introduction of weaker A-B and B-B interactions disrupt these pathways, leading to a fragmentation of the dry phase into smaller, more numerous, and less connected bubbles (Fig. SI26-d). The smoother evolution of bubble statistics across pressure in this regime indicates a gradual breakdown of long-range cooperativity. At high B concentrations (>15%), the network becomes fully fragmented, and intrusion proceeds through disordered, weakly correlated local events. This regime is characterised by persistently small, disconnected bubbles and low connectivity (Fig. SI26b-d), consistent with the loss of collective switching behaviour. Moreover, the higher noise and fluctuations in the curves at high B levels (Fig. SI26a-c) suggest that the process becomes more irregular and locally driven, as collective transitions give way to isolated events. These observations align with the experimental results, confirming that even minimal chemical heterogeneity introduced by bIm linker can significantly disrupt the cooperative wetting dynamics of the framework. Fig. SI27 reveals that increasing the $\omega_{\text{w}}^{\text{A}}$ generally elevates both P_{int} and P_{ext}, reflecting stronger resistance to liquid intrusion and extrusion. Systems with higher $\omega_{\text{w}}^{\text{A}}$ (e.g., 12 or 13) display a consistent upward shift in both pressures, while increasing $\omega_{\text{w}}^{\text{B}}$ (from 15 to 16) further enhances P_{ext}. The I_{AA} modulates the cooperative coupling among A cages: larger I_{AA} values amplify the collective response, leading to higher pressures and broader hysteresis loops. In particular, the system with I_{AA} = 1.1 exhibits the most pronounced hysteresis, highlighting how stronger A–A cooperativity promotes asymmetric energy landscapes and delays extrusion. Conversely, weaker coupling (I_{AA} = 0.85) produces narrower hysteresis and lower transition pressures, consistent with a more independent cage filling mechanism. Further, stronger I_{AB} (0.7 or 0.95) coupling preserves cooperative transitions between A and B domains, yielding higher pressures and broader hysteresis, while weaker I_{AB} (0.3) leads to fragmented, less correlated responses. Overall, barrier asymmetry primarily tunes the absolute pressure scale, while I_{AA} and I_{AB} govern the degree of cooperativity and hysteretic behaviour across mIm/bIm compositions in ZIF-7-8.

Furthermore, Figs. SI28 to SI29 present a side-by-side comparison of intrusion and extrusion processes across varying B content, revealing how compositional heterogeneity affects the wetting and drying. At 0% B, the system exhibits sharp, cooperative transitions: nearly all A cages switch state within a narrow pressure range, resulting in pronounced hysteresis. As B content increases to 3% and 5%, intrusion and extrusion fronts become less abrupt and increasingly spatially localised, with B

cages appearing and influencing the surrounding A cage behaviour. At 10% B, transitions are highly irregular, with both A and B cages filling or emptying in scattered clusters. Overall, increasing B content disrupts the system's collective behaviour, transitioning it from a strongly coupled to a weakly coupled regime with independent wetting and drying events.

Fig. SI30 illustrates the mechanistic shift in intrusion/extrusion behaviour as B content increases. The top row reveals that while both $P_{\text{int}}\ and\ P_{\text{ext}}$ increase with B%, $\langle \Delta P \rangle$ decreases significantly $(R^2 = 0.66)$, reflecting a collapse in hysteresis. This collapse is further captured by the boxplots in the middle row, which show a steep drop in hysteresis between 5-10% B, followed by a plateau. The 3D scatter plot highlights a strong clustering of low Δ and low hysteresis at high B content, emphasising a decoupling of wetting/drying transitions (i.e., the system no longer exhibits large pressure differences between intrusion and extrusion, nor wide hysteresis loops). Most critically, the segmented slope analysis in the bottom row confirms behaviour: the low B regime shows a sharp negative slope in ΔP (-0.2 MPa/%) and strong hysteresis correlation $(R^2 > 0.95)$, consistent with cooperative switching. In contrast, the mid and high B regimes show flattened or even reversed ΔP slopes, indicating fragmentation of cooperative domains and potential emergence of localised or B-type domain responses. These findings align closely with the breakdown of A-A cage interactions in the network model.

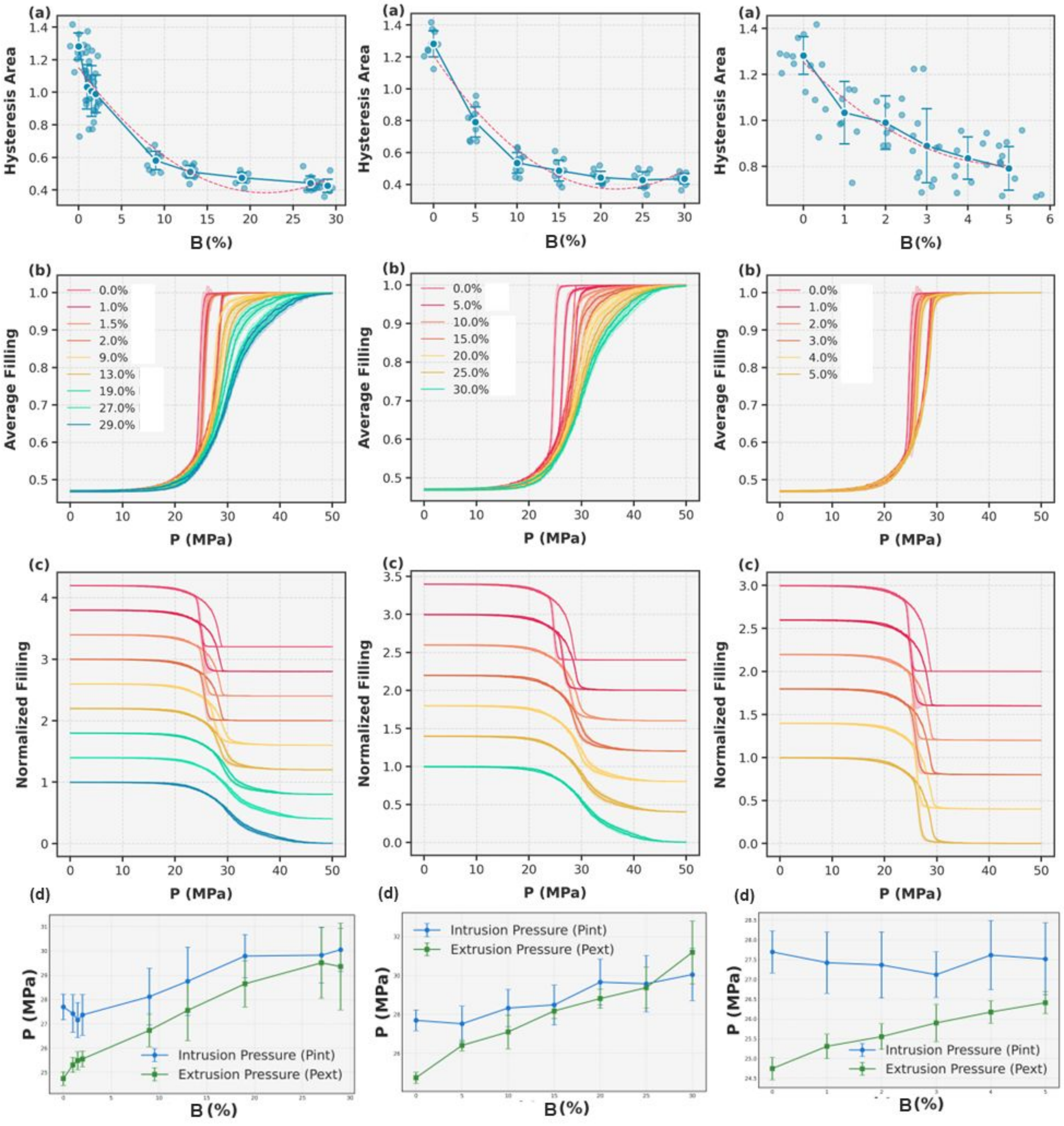

Figure SI20. (a) Hysteresis versus B-type cage content. (b) Intrusion-extrusion curves: hysteresis at different B%. (c) Normalised filling as a function of pressure. (d) Intrusion (P_{int}) and extrusion (P_{ext}) pressures as a function of B concentration. Parameters: $\boldsymbol{\omega_w}^{\mathbf{A}} = \mathbf{11}$, $\boldsymbol{\omega_d}^{\mathbf{A}} = \mathbf{2}$, $\boldsymbol{\omega_w}^{\mathbf{B}} = \mathbf{15}$, $\boldsymbol{\omega_d}^{\mathbf{B}} = \mathbf{3}$, $\boldsymbol{v_w}^{\mathbf{A}} = \mathbf{0.24}$, $\boldsymbol{v_d}^{\mathbf{A}} = \mathbf{0.06}$, $\boldsymbol{v_w}^{\mathbf{B}} = \mathbf{0.2}$, $\boldsymbol{v_d}^{\mathbf{A}} = \mathbf{0.04}$, $\mathbf{I_{AA}} = \mathbf{0.85}$, $\mathbf{I_{AB}} = \mathbf{I_{BA}} = \mathbf{0.5}$, $\mathbf{I_{BB}} = \mathbf{0.01}$.

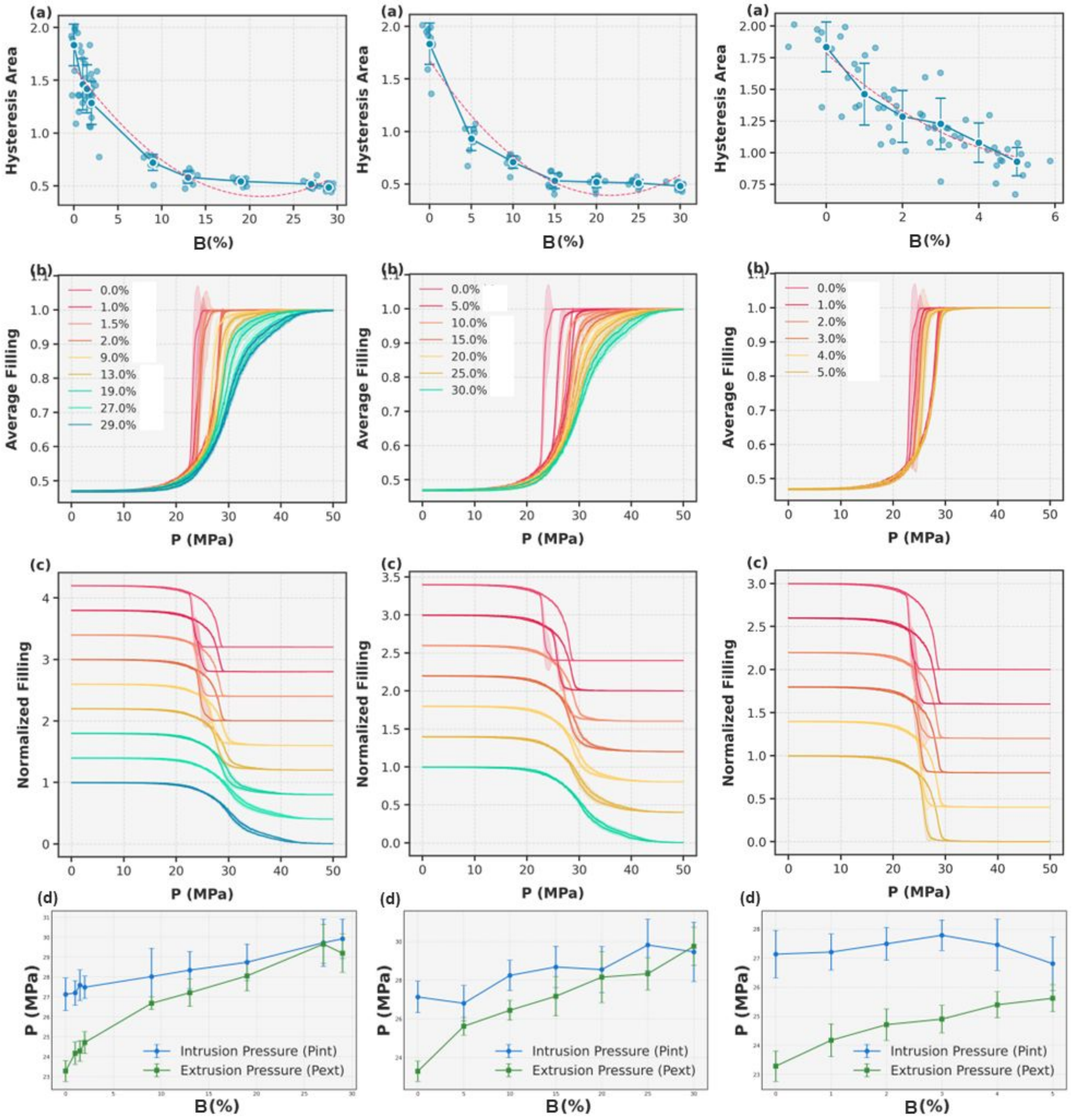

Figure SI21. (a) Hysteresis versus B-type cage content. (b) Intrusion-extrusion curves: hysteresis at different B%. (c) Normalised filling as a function of pressure. (d) Intrusion (P_{int}) and extrusion (P_{ext}) pressures as a function of B concentration. Parameters: $\boldsymbol{\omega_w}^{\mathbf{A}} = \mathbf{11}$, $\boldsymbol{\omega_d}^{\mathbf{A}} = \mathbf{2}$, $\boldsymbol{\omega_w}^{\mathbf{B}} = \mathbf{15}$, $\boldsymbol{\omega_d}^{\mathbf{B}} = \mathbf{3}$, $\boldsymbol{v_w}^{\mathbf{A}} = \mathbf{0.24}$, $\boldsymbol{v_d}^{\mathbf{A}} = \mathbf{0.06}$, $\boldsymbol{v_w}^{\mathbf{B}} = \mathbf{0.2}$, $\boldsymbol{v_d}^{\mathbf{A}} = \mathbf{0.04}$, $\mathbf{I_{AA}} = \mathbf{0.95}$, $\mathbf{I_{AB}} = \mathbf{I_{BA}} = \mathbf{0.5}$, $\mathbf{I_{BB}} = \mathbf{0.01}$.

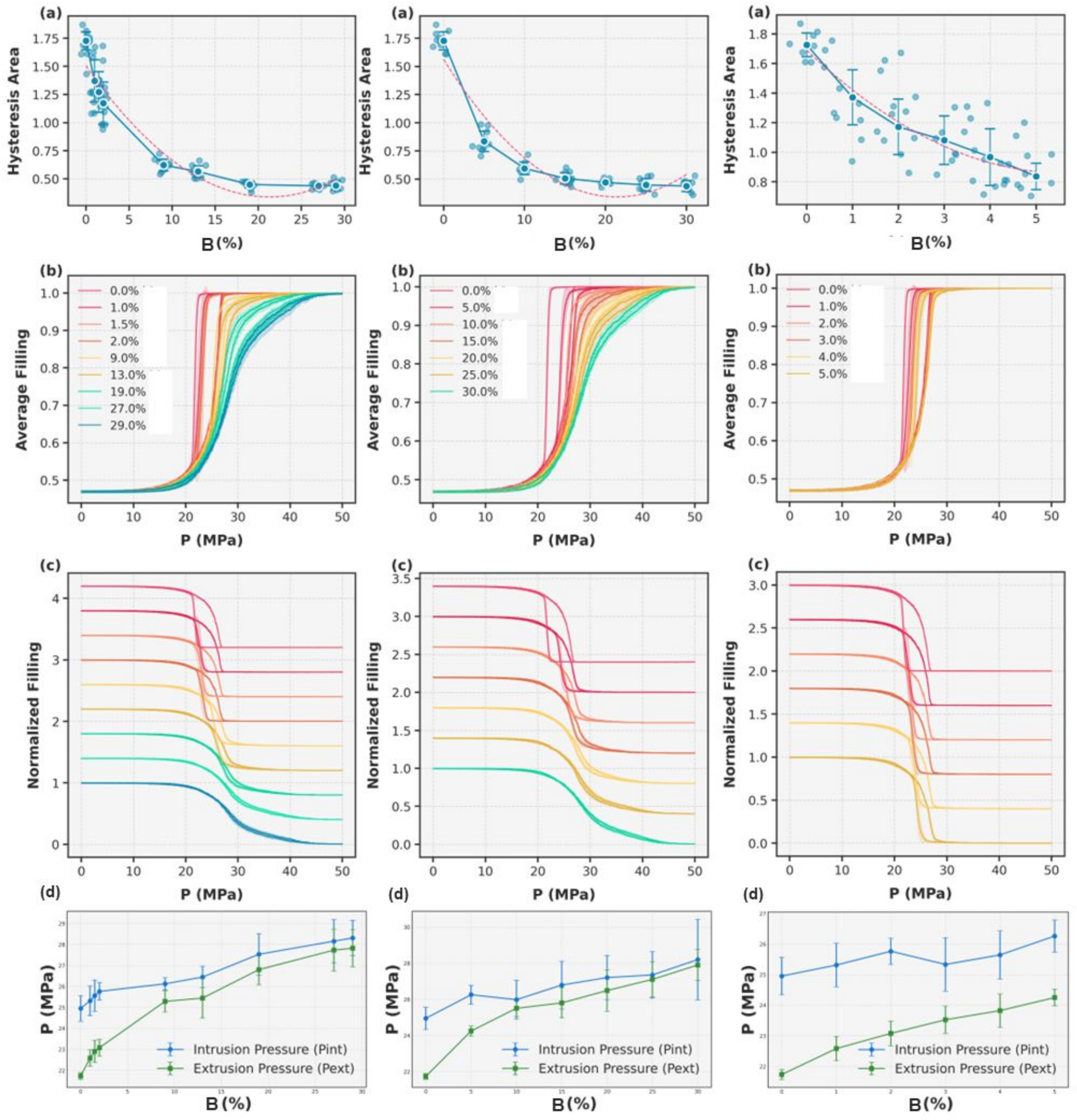

Figure SI22. (a) Hysteresis versus B-type cage content. (b) Intrusion-extrusion curves: hysteresis at different B%. (c) Normalised filling as a function of pressure. (d) Intrusion (P_{int}) and extrusion (P_{ext}) pressures as a function of B concentration. Parameters: $\boldsymbol{\omega_{\mathrm{w}}}^{\mathbf{A}} = \mathbf{11}$, $\boldsymbol{\omega_{\mathrm{d}}}^{\mathbf{A}} = \mathbf{2}$, $\boldsymbol{\omega_{\mathrm{w}}}^{\mathbf{B}} = \mathbf{15}$, $\boldsymbol{\omega_{\mathrm{d}}}^{\mathbf{B}} = \mathbf{3}$, $\boldsymbol{v_{\mathrm{w}}}^{\mathbf{A}} = \mathbf{0.26}$, $\boldsymbol{v_{\mathrm{d}}}^{\mathbf{A}} = \mathbf{0.06}$, $\boldsymbol{v_{\mathrm{w}}}^{\mathbf{B}} = \mathbf{0.2}$, $\boldsymbol{v_{\mathrm{d}}}^{\mathbf{A}} = \mathbf{0.04}$, $\mathbf{I_{AA}} = \mathbf{0.95}$, $\mathbf{I_{AB}} = \mathbf{I_{BA}} = \mathbf{0.5}$, $\mathbf{I_{BB}} = \mathbf{0.01}$.

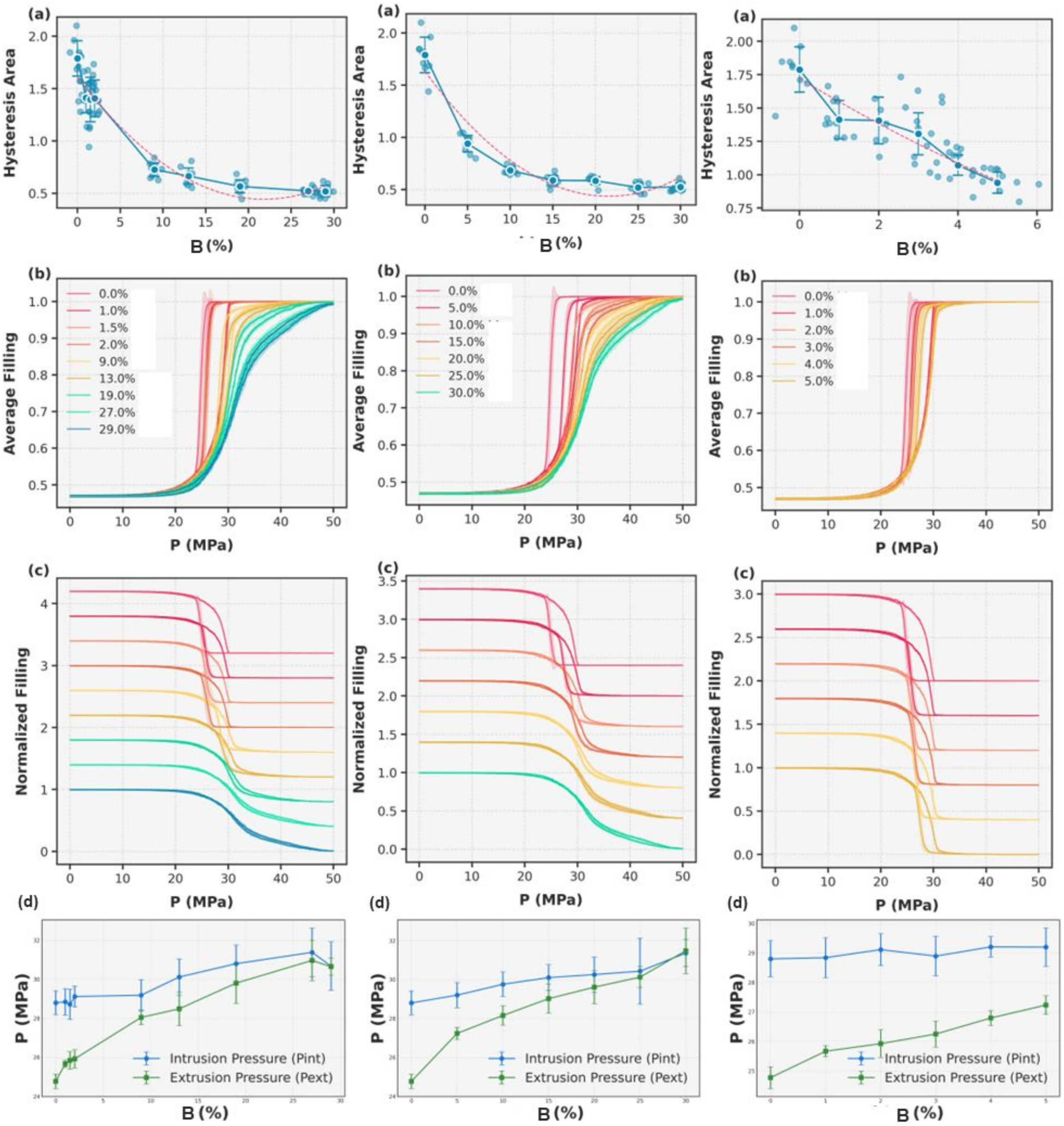

Figure SI23. (a) Hysteresis versus B-type cage content. (b) Intrusion-extrusion curves: hysteresis at different B%. (c) Normalised filling as a function of pressure. (d) Intrusion (P_{int}) and extrusion (P_{ext}) pressures as a function of bIm linker concentration. Parameters: $\boldsymbol{\omega_w}^{\mathbf{A}} = \mathbf{11}$, $\boldsymbol{\omega_d}^{\mathbf{A}} = \mathbf{2}$, $\boldsymbol{\omega_w}^{\mathbf{B}} = \mathbf{16}$, $\boldsymbol{\omega_d}^{\mathbf{B}} = \mathbf{3}$, $\boldsymbol{\upsilon_w}^{\mathbf{A}} = \mathbf{0.26}$, $\boldsymbol{\upsilon_d}^{\mathbf{A}} = \mathbf{0.06}$, $\boldsymbol{\upsilon_w}^{\mathbf{B}} = \mathbf{0.2}$, $\boldsymbol{\upsilon_d}^{\mathbf{A}} = \mathbf{0.04}$, $\mathbf{I_{AA}} = \mathbf{0.95}$, $\mathbf{I_{AB}} = \mathbf{I_{BA}} = \mathbf{0.5}$, $\mathbf{I_{BB}} = \mathbf{0.01}$.

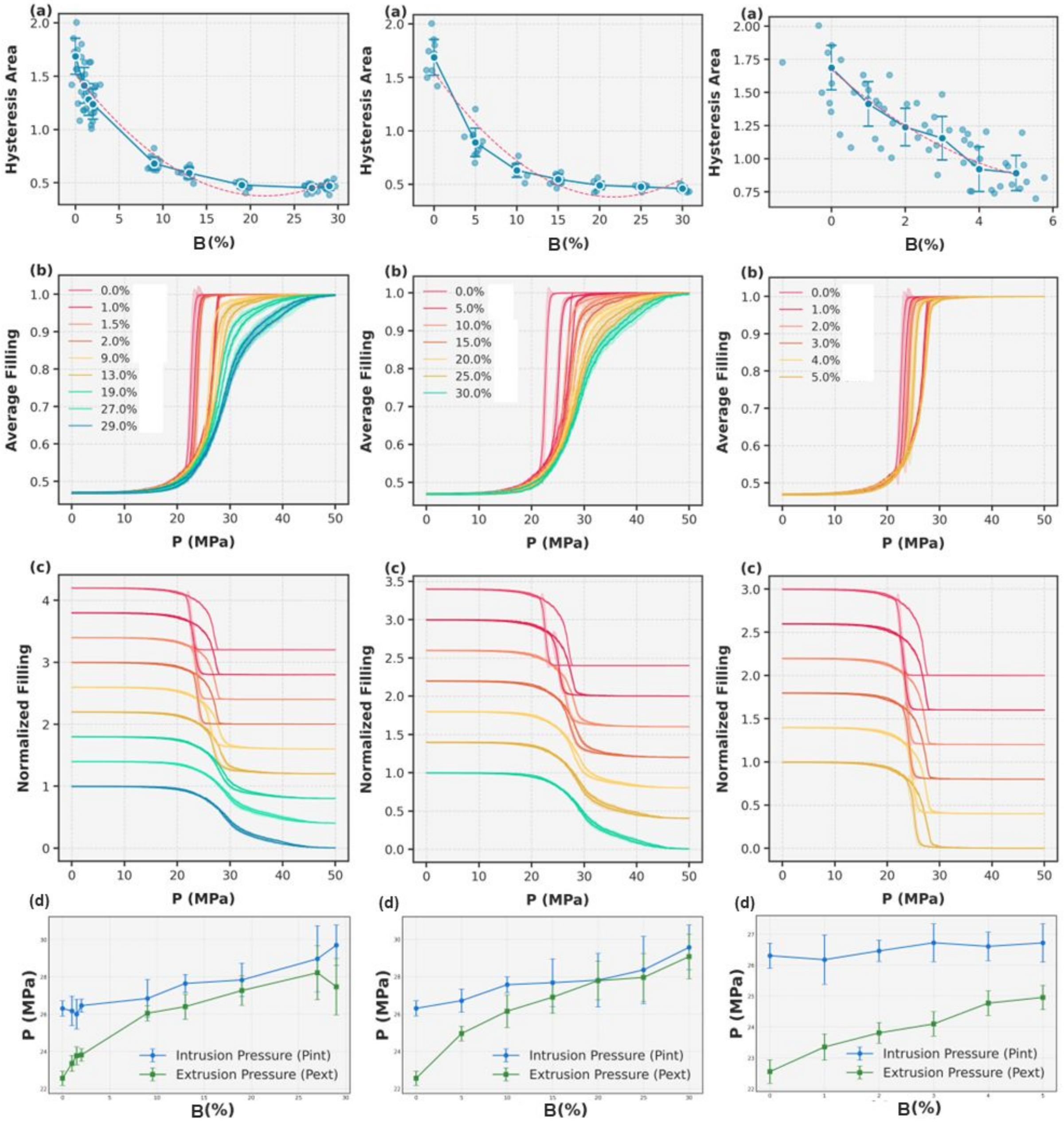

Figure SI24. (a) Hysteresis versus B-type cage content. (b) Intrusion-extrusion curves: hysteresis at different B%. (c) Normalised filling as a function of pressure. (d) Intrusion (P_{int}) and extrusion (P_{ext}) pressures as a function of bIm linker concentration. Parameters: $\boldsymbol{\omega_w}^{\mathbf{A}} = \mathbf{11}$, $\boldsymbol{\omega_d}^{\mathbf{A}} = \mathbf{2}$, $\boldsymbol{\omega_w}^{\mathbf{B}} = \mathbf{15}$, $\boldsymbol{\omega_d}^{\mathbf{B}} = \mathbf{3}$, $\boldsymbol{v_w}^{\mathbf{A}} = \mathbf{0.25}$, $\boldsymbol{v_d}^{\mathbf{A}} = \mathbf{0.06}$, $\boldsymbol{v_w}^{\mathbf{B}} = \mathbf{0.19}$, $\boldsymbol{v_d}^{\mathbf{A}} = \mathbf{0.04}$, $\mathbf{I_{AA}} = \mathbf{0.95}$, $\mathbf{I_{AB}} = \mathbf{I_{BA}} = \mathbf{0.5}$, $\mathbf{I_{BB}} = \mathbf{0.01}$.

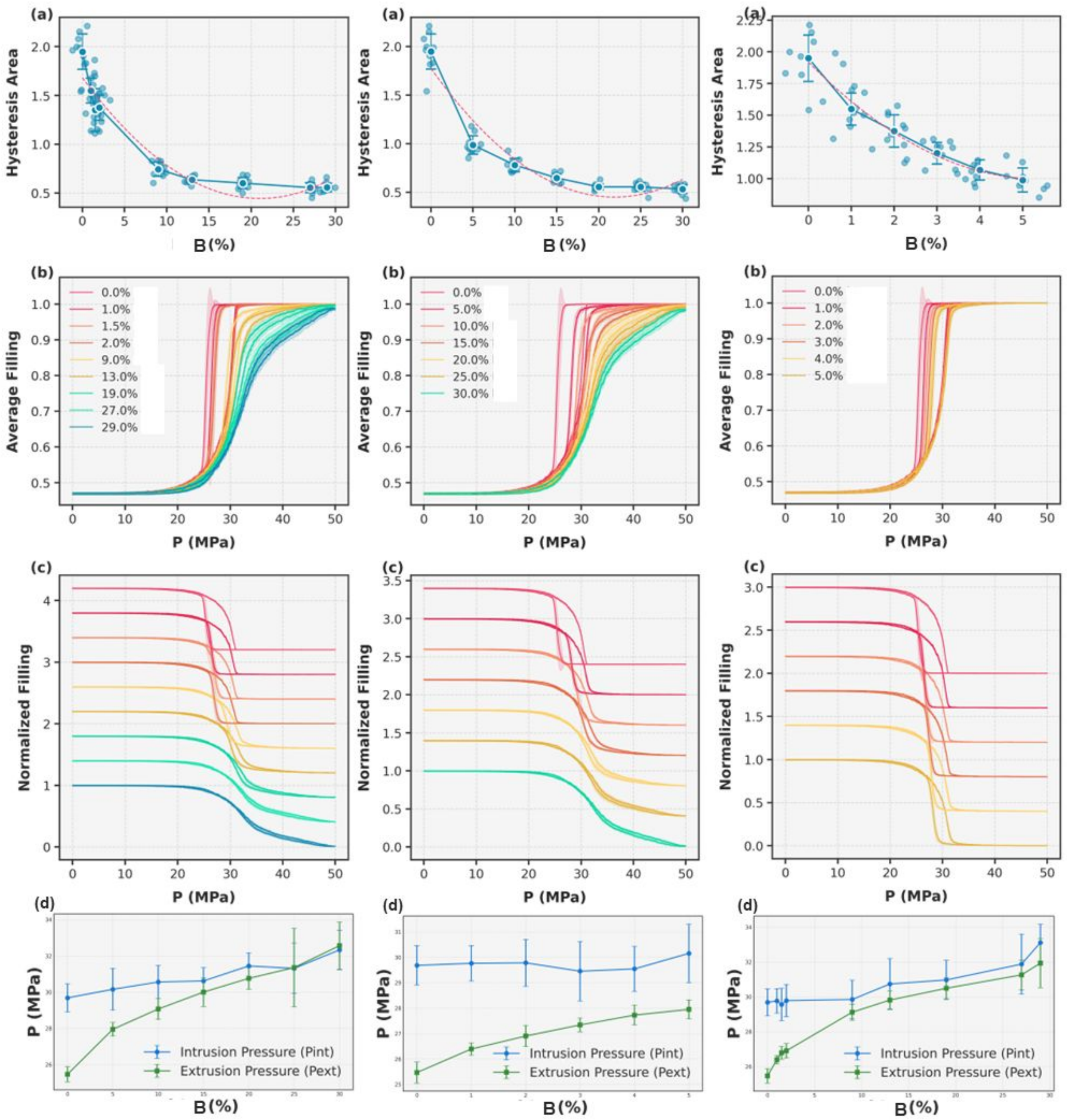

Figure SI25. (a) Hysteresis versus B-type cage content. (b) Intrusion-extrusion curves: hysteresis at different B%. (c) Normalised filling as a function of pressure. (d) Intrusion (P_{int}) and extrusion (P_{ext}) pressures as a function of bIm linker concentration. Parameters: $\boldsymbol{\omega_w}^{\mathbf{A}} = \mathbf{12}$, $\boldsymbol{\omega_d}^{\mathbf{A}} = \mathbf{2}$, $\boldsymbol{\omega_w}^{\mathbf{B}} = \mathbf{16}$, $\boldsymbol{\omega_d}^{\mathbf{B}} = \mathbf{3}$, $\boldsymbol{v_w}^{\mathbf{A}} = \mathbf{0.25}$, $\boldsymbol{v_d}^{\mathbf{A}} = \mathbf{0.06}$, $\boldsymbol{v_w}^{\mathbf{B}} = \mathbf{0.19}$, $\boldsymbol{v_d}^{\mathbf{A}} = \mathbf{0.04}$, $\mathbf{I_{AA}} = \mathbf{0.95}$, $\mathbf{I_{AB}} = \mathbf{I_{BA}} = \mathbf{0.5}$, $\mathbf{I_{BB}} = \mathbf{0.01}$.

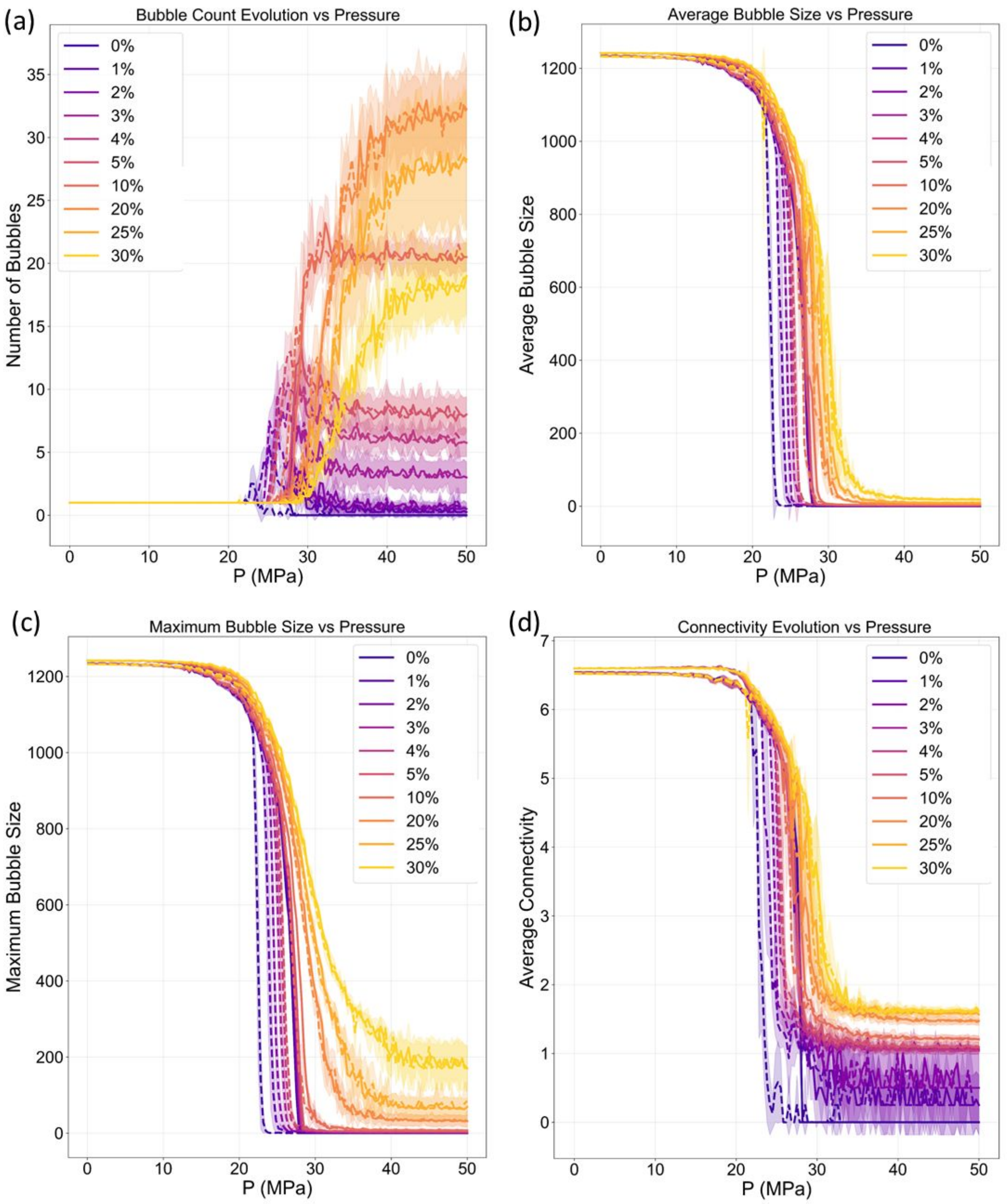

Figure SI26. Evolution of dry bubble characteristics during intrusion in ZIF-8 and ZIF-7-8 with increasing B-type cage content (0-30%). Dry bubbles are defined as connected clusters of dry nodes and tracked over time. The algorithm detects bubbles via breadth-first search and characterises them by size, shape, and connectivity. To track bubbles across timesteps, it employs a multi-criteria cost function and solves the optimal assignment using the Hungarian algorithm. (a) Number of bubbles vs. pressure. (b) Average bubble size vs. pressure. (c) Maximum bubble size vs. pressure. (d) Average connectivity of nodes within bubbles vs. pressure. Shaded bands indicate standard deviation.

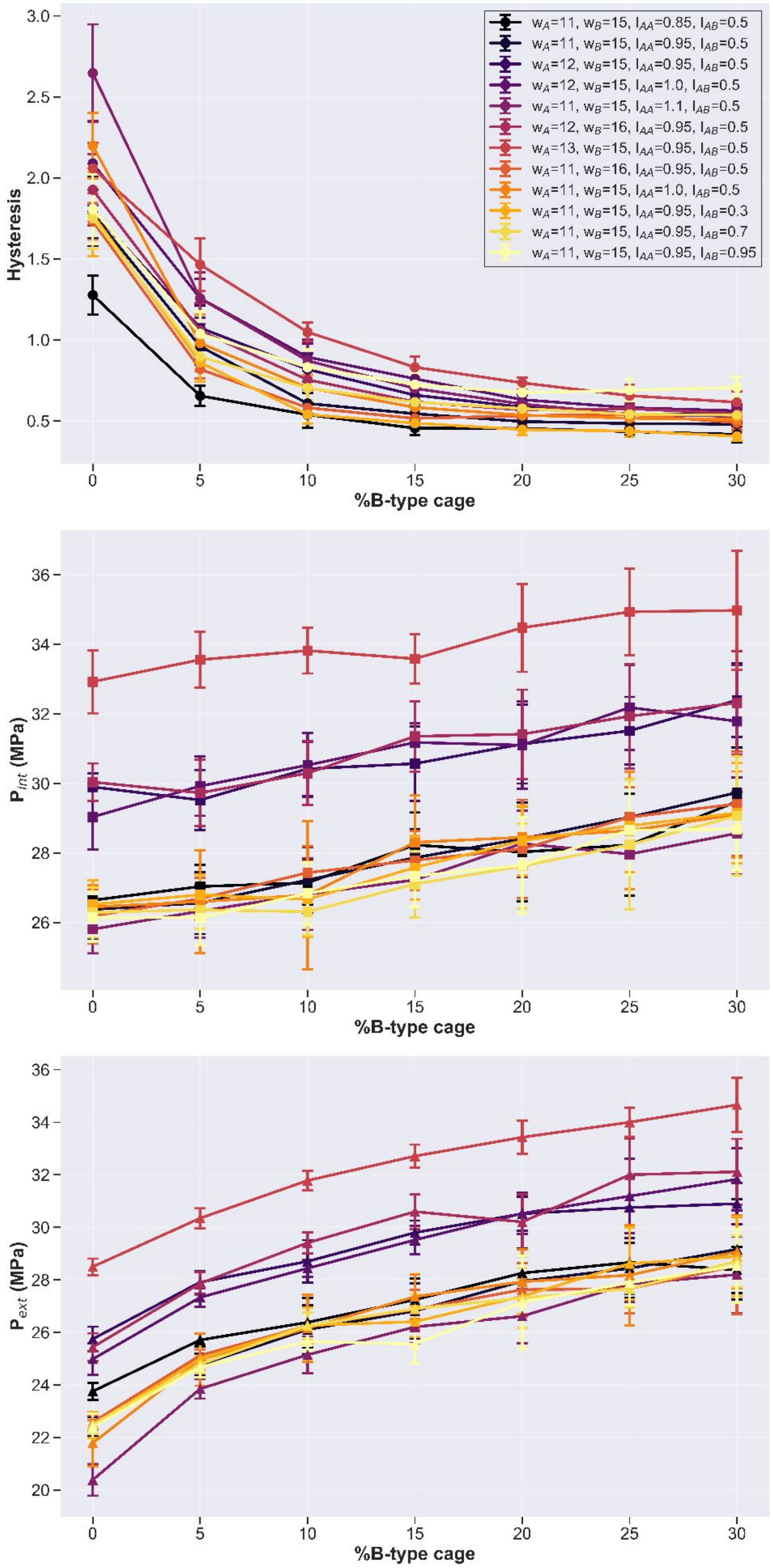

Figure SI27. Evolution of (a) average intrusion pressure, (b) extrusion pressure, (c) hysteresis width with respect to the %B-type cage content. Each

curve corresponds to a distinct parameter set (ω_w^A,ω_w^B,I_{AA}), as indicated in the legend.

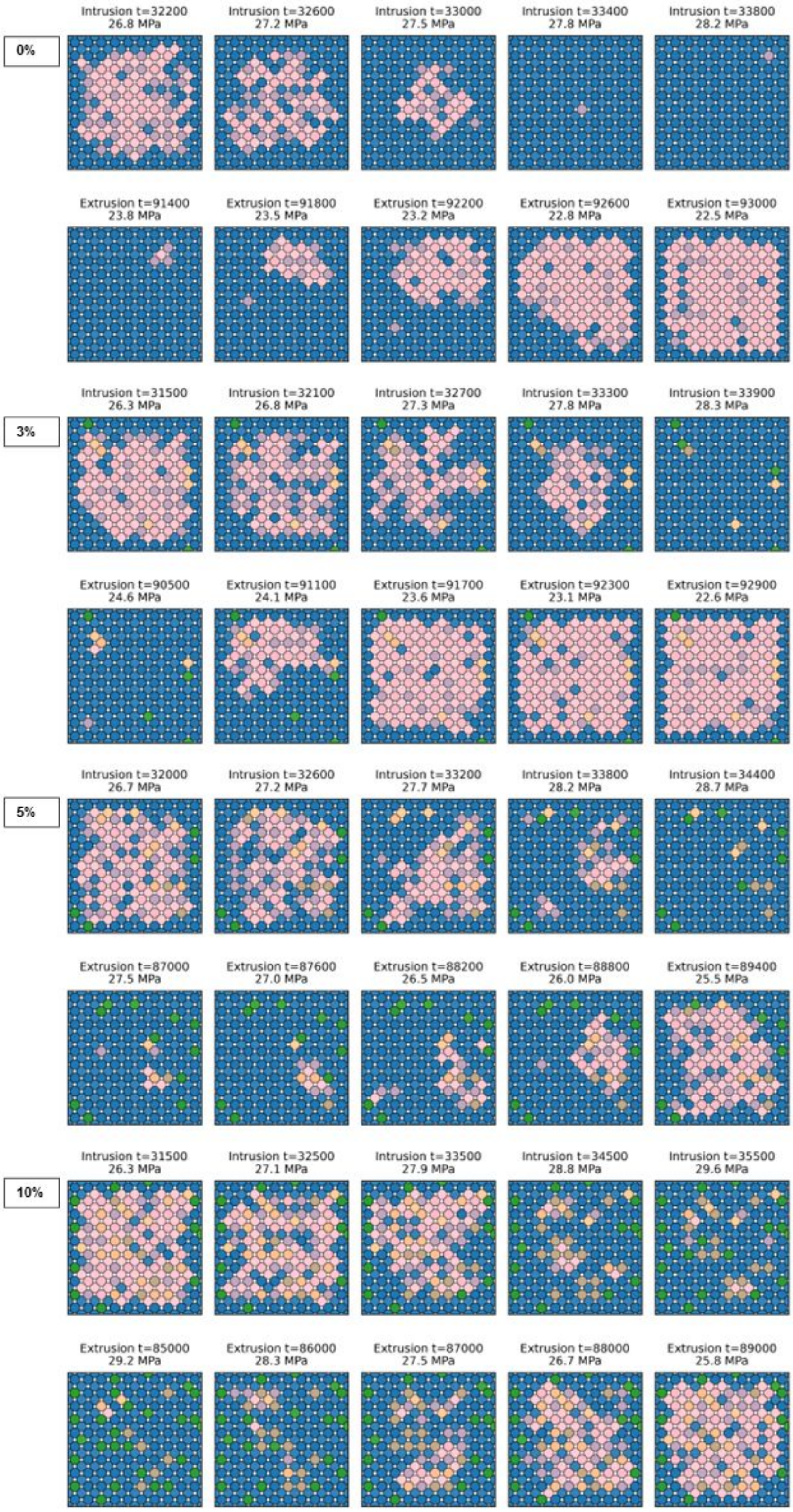

Figure SI28. Two-dimension (XY-plane) snapshots of fluid intrusion (top rows) and extrusion (bottom rows) in a ZIF-7-8 topology-like framework (0, 3, 5 and

10% B) during a pressure cycle. Colours represent cage states: filled/empty A-type and B-type cages. Timesteps (t) and corresponding pressure (MPa) are labelled for each frame, showing the progression of wetting and drying processes.

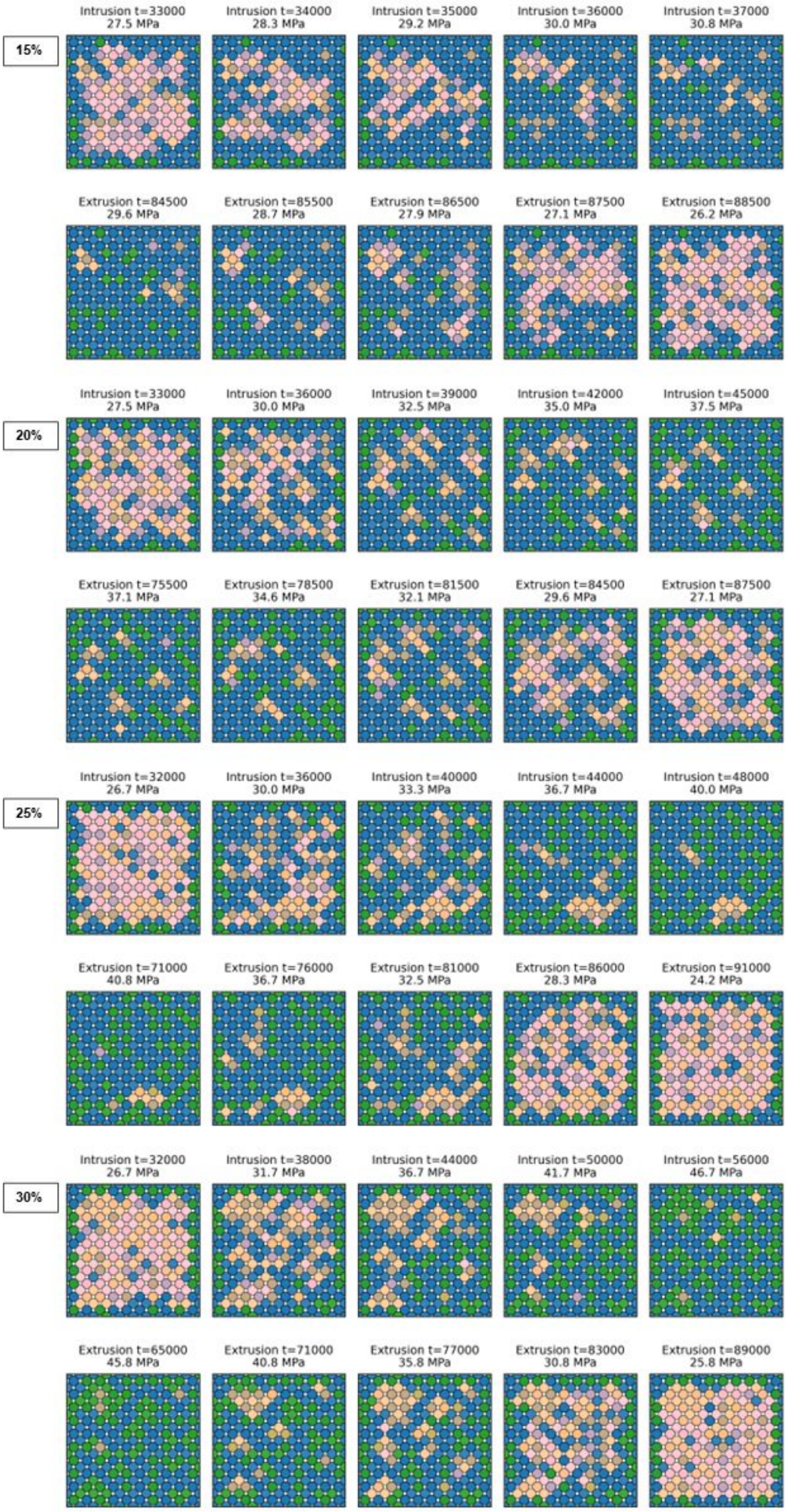

Figure SI29. Two-dimension (XY-plane) snapshots of fluid intrusion (top rows) and extrusion (bottom rows) in a ZIF-7-8 topology like framework (15, 20, 25 and 30% bIm) during a pressure cycle. Colours represent cage states: filled/empty A-type and B-type cages. Timestep (t) and corresponding pressure (MPa) are labelled for each frame, showing the progression of wetting and drying processes.

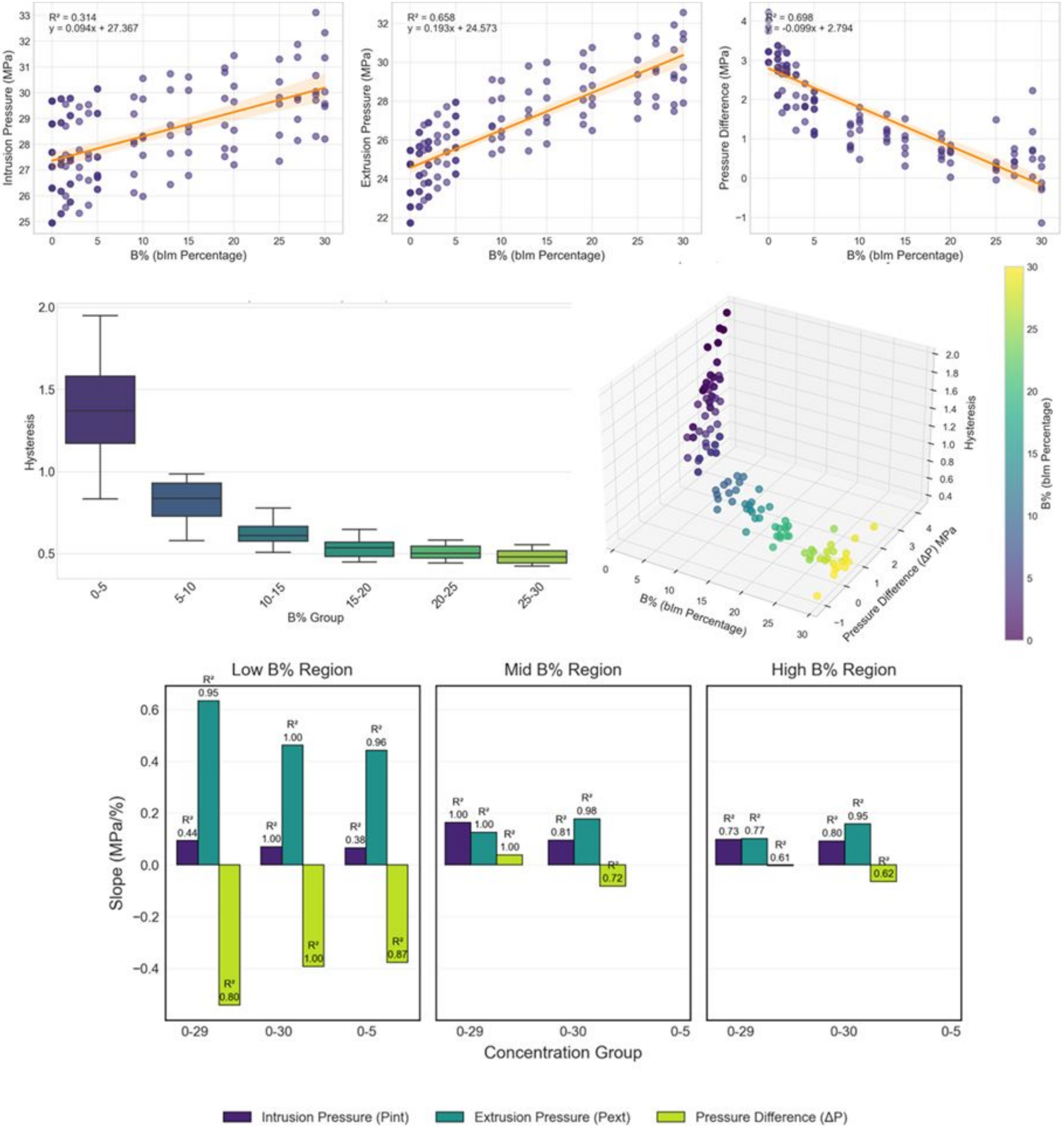

Figure SI30. Compositional dependence of $P_{\text{int}}\, and\, P_{\text{ext}}$ and pressure difference ($\Delta P = P_{\text{int}} - P_{\text{ext}}$) as a function of bIm content in mixed-linker ZIF systems. Top row: scatter plots and linear fits show P_{int} (left), P_{ext} (middle), and ΔP(right) trends with increasing B%. Middle row: (Left) Hysteresis narrowing shown via boxplots across bIm% groups. (Right) 3D scatter plot showing correlation between B-type cage %, ΔP, and hysteresis. Bottom row: slopes of pressure trends across low (0%-5%), mid (5%-15%), and high (15%-30%) B-

type cage regimes, confirming two-regime behavior in ΔP and hysteresis. Correlation coefficients (R^2) denote linearity within each region.

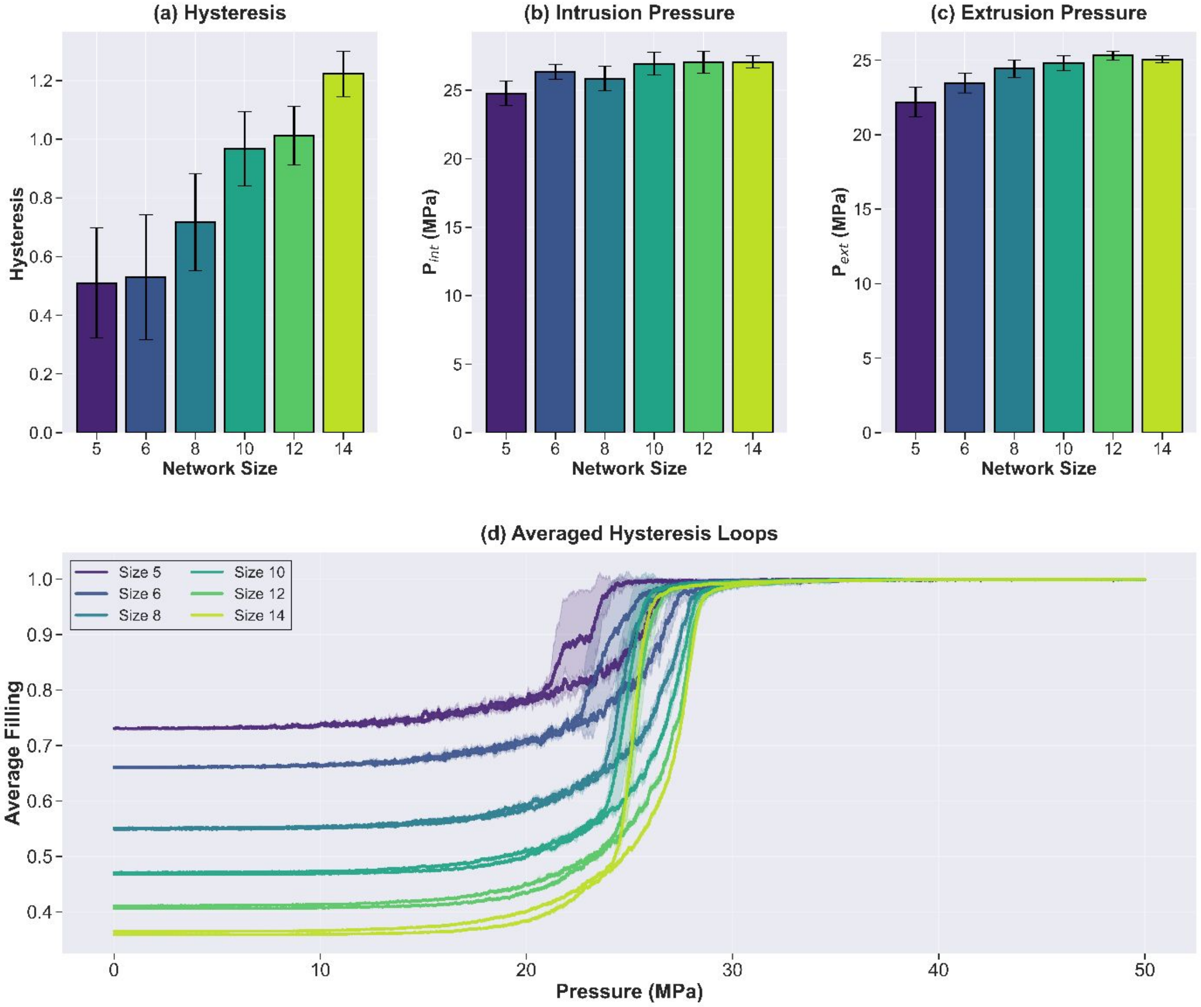

Figure SI31. Network size affecting the intrusion-extrusion behaviour of ZIF-7-8 topology with 5% of B-type cages across different growth: 5 (341 nodes), 6 (559 nodes), 8 (1241 nodes), 10 (2331 nodes), 12 (3925 nodes), and 14 (6119 nodes). Both intrusion/extrusion pressures and hysteresis increase systematically with network size, reflecting a transition from surface-dominated to bulk-dominated behaviour. Smaller networks exhibit gradual, stochastic filling due to pre-filled surface cages, while larger networks display sharp, cooperative transitions characteristic of true bulk behaviour.

To investigate the role of linker homogeneity in the collective wetting behavior of ZIF-7-8, we explored different arrangements of bIm linkers within the stochastic framework

model. In this context, homogeneity can refer to two aspects: (i) the spatial distribution of modified cages within the crystallite and (ii) the statistical distribution of bIm linkers across cages, i.e., whether the same total number of bIm linkers is distributed across many cages or concentrated in a smaller number of cages.

Spatial distribution of B-type cages within the crystallite

First, we examined how the spatial arrangement of B-type cages affects the intrusion–extrusion behavior. In the model, B-type cages represent cages containing bIm linkers with modified wetting properties compared to A-type cages. Three spatial configurations were considered: B-type cages located preferentially at the surface of the crystallite, at its center, or uniformly distributed throughout the structure.

The results are shown in Fig. SI32. Only a uniform distribution of B-type cages reproduces the experimentally observed behaviour, namely single-step intrusion and extrusion transitions with a progressive reduction of hysteresis. In contrast, clustering B-type cages at the surface or center leads to features not observed experimentally, including two-step intrusion–extrusion processes at higher fractions of modified cages and unrealistic hysteresis values. These results suggest that the experimental trends are consistent with a statistically uniform spatial distribution of modified cages within the crystallite.

Statistical distribution of bIm linkers across cages

We then examined how the distribution of bIm linkers across cages influences the collective wetting response. For a given overall linker concentration, bIm linkers could either be distributed uniformly across many cages or concentrated in fewer cages. Because the benzene ring of bIm increases cage hydrophobicity, multiple bIm linkers in the same cage are expected to increase the wetting barrier.

To test this effect while keeping the total number of bIm linkers constant, we reduced the fraction of B-type cages while increasing their hydrophobicity, modeled through the wetting barrier parameter $\boldsymbol{w_b}$. Specifically, we compared the homogeneous case (one bIm per cage, 30% B-type cages, $\boldsymbol{w_b = 15}$) with scenarios corresponding to multiple bIm per cage: 2 (15%, $\boldsymbol{w_b = 18}$), 4 (7.5%, $\boldsymbol{w_b = 21}$), and 8 (3.75%, $\boldsymbol{w_b = 23}$). A reference case with constant $\boldsymbol{w_b = 15}$ was also considered.

The results (Fig. SI33) show that concentrating bIm linkers into fewer cages leads to intrusion–extrusion curves where hysteresis is only weakly affected, in contrast with the experimental trends and the model predictions reported in the main text. This behaviour is observed both when the wetting barrier is kept constant and when it is increased to account for multiple bIm linkers per cage. These results indicate that the collective wetting behaviour is primarily governed by the number of modified cages rather than the magnitude of the hydrophobicity change of individual cages.

Overall, the simulations indicate that the experimental behaviour is best reproduced by a statistically homogeneous distribution of bIm linkers across cages, corresponding

to the largest number of modified cages with moderate changes in individual cage hydrophobicity.

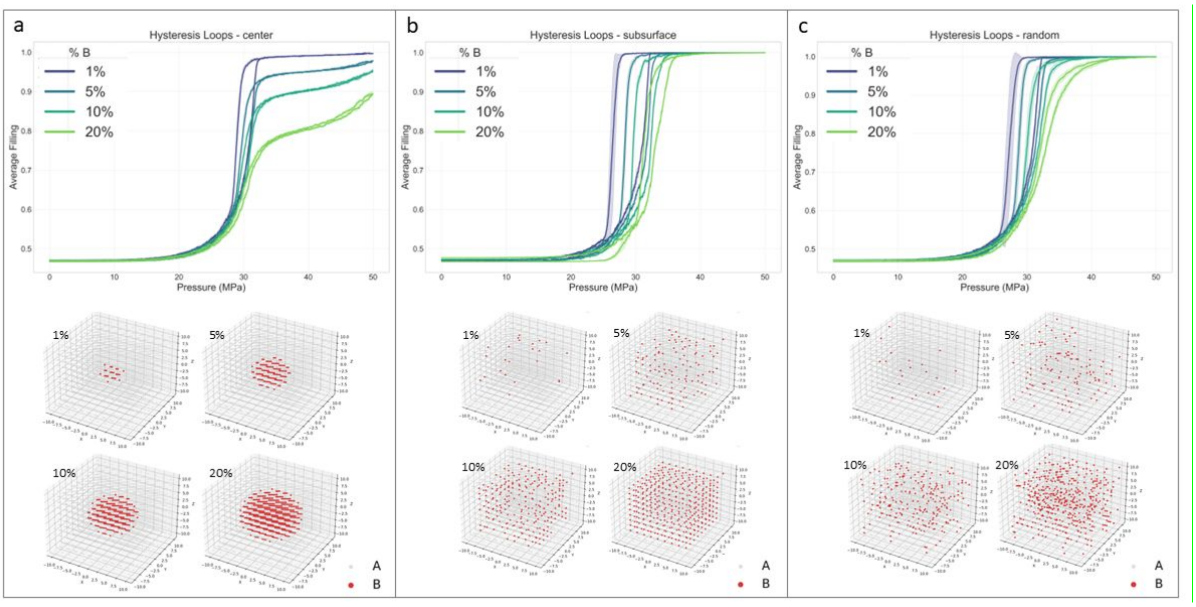

Figure SI32. Stochastic model simulation of intrusion-extrusion behavior at different B-cage concentrations and locations in the SOD framework: (a) center, (b) subsurface, and (c) random.

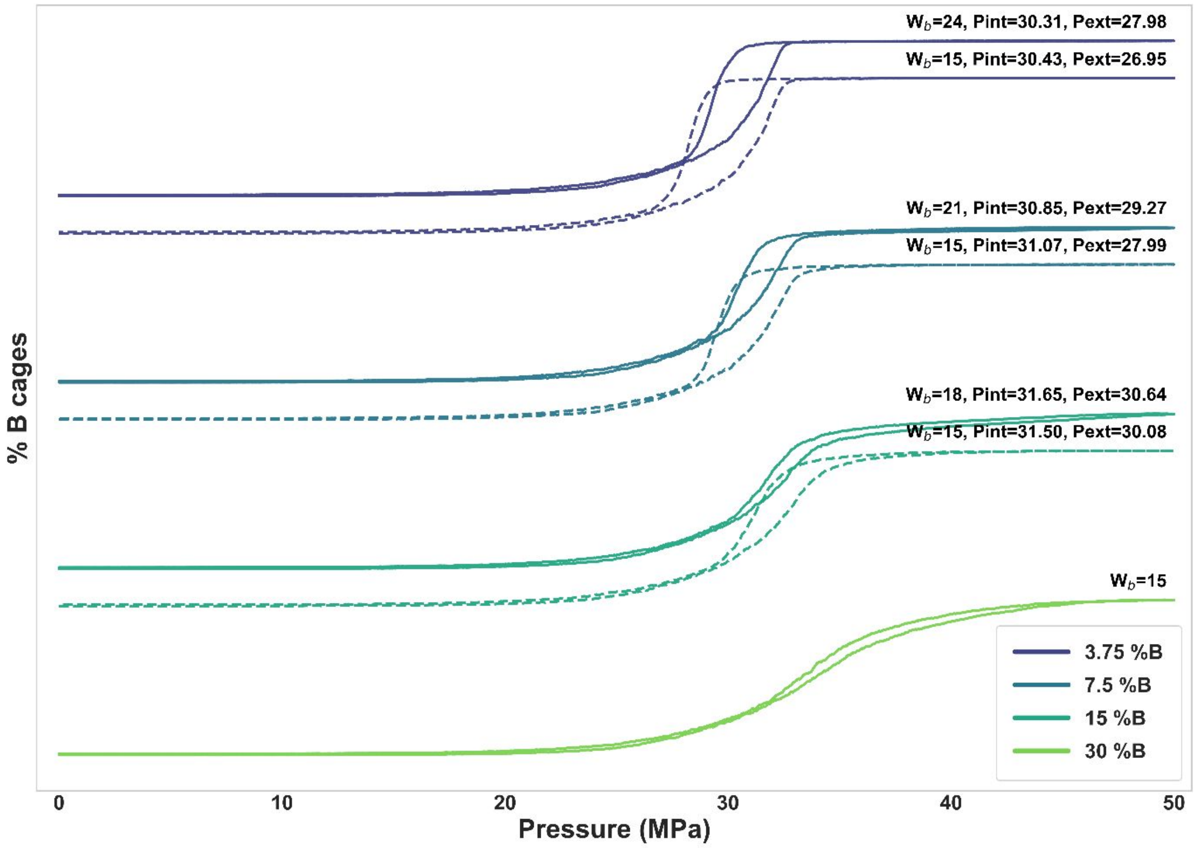

Figure SI33. Effect of the number of bIm per cage when the total number of bIm is kept constant. Results are obtained by simulating with the stochastic model the intrusion-extrusion behavior of SOD mixed-linker framework decreasing the fraction of type-B cages while increasing the wetting barrier (w_b), to mimic the effect of multiple bIm per cage. For reference, the system in which the wetting barrier is constant at the single bIm per cage level is reported (w_b=15, dashed lines).

SI9. Particle size distribution analysis

SEM measurements were performed on a Thermo Fisher Quanta 200FEG. Details on the working conditions are reported in Fig. SI34.

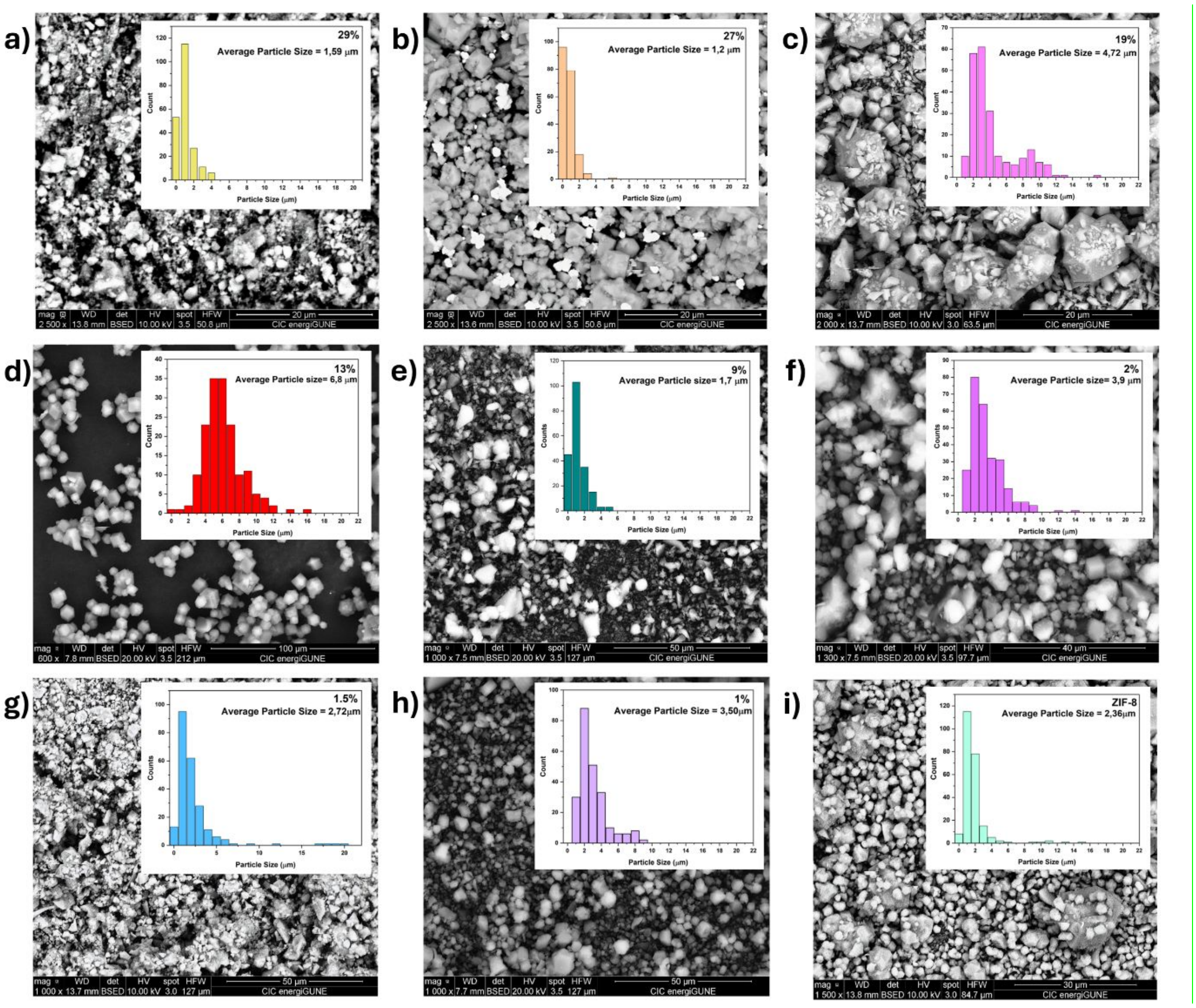

Figure SI34. SEM images and particle size distributions of ZIF-7-8 samples with a) 29%, b)27%, c)17%, d)13%, e)9%, f)2%, g)1.5%, h)1%, and i) 0% bIm concentration.

SI10. Theoretical analysis of bIm linkers distribution in a ZIF-7-8 crystallite

We investigate whether the minority bIm linker inserted into the mixed-linker MOF is uniformly distributed throughout the sample, or whether it accumulates in some cages, leading to a non-trivial probability distribution for the number of bIm linkers per cage. Because this question cannot be readily addressed experimentally, we formulate it theoretically (as discussed in detail below), using input parameters obtained from *ab initio* density functional theory simulations.

Even within a theoretical framework, however, a fully general treatment is highly challenging. We therefore focus on the following concrete equilibrium (thermodynamic) question. For ZIF-7-8 at a fixed nominal bIm composition, namely 3% - corresponding to one bIm per ZIF-7-8 unit cell - is it more likely for the linker to be distributed uniformly (case I: one bIm per unit cell), or to form a non-uniform pattern in which half of the unit cells contain two bIm linkers while the remaining half are ZIF-8-like (case II)? We address this question within constant-temperature statistical mechanics. The key quantity is the equilibrium probability ratio P_I/P_{II}, where P_{x} is the probability of case $\mathrm{X} \in \{I, II\}$. Under isothermal conditions, this ratio is determined by configurational Boltzmann weights, i.e., by the ratio of the corresponding configurational integrals.

$$\frac{P_{II}}{P_I} = \frac{\int_{\Omega_{\mathrm{II}}} dR\, exp(-\beta V(R))}{\int_{\Omega_{\mathrm{I}}} dR\, exp(-\beta V(R))} \qquad (1)$$

Where Ω_x is the 3N-dimensional configuration space consistent with case X, $V(R)$ is the potential energy, and $\beta = 1/k_B T$.

Generally, these integrals cannot be evaluated in closed form. However, by introducing suitable, physically motivated approximations (detailed below), we derive the following semi-analytical expression for the relative probability of the two states:

$$\frac{P_{II}}{P_I} = \sqrt{\frac{2}{\pi N}} \cdot \exp\left(-\frac{{}^{N}\!/_{2} \cdot \Delta V_0 - N\, k_B T\, \ln\sqrt{{}^{23}\!/_{12}}}{k_B T}\right) \qquad (2)$$

where N is the number of unit cells in the crystallite and $\Delta V_0 = \mathrm{V}(R_{\mathrm{II}}^0) - \mathrm{V}(R_{\mathrm{I}}^0)$ is the difference in potential energy between the two representative minima R_0 associated with states II and I, respectively.

For sufficiently large crystallites (large N), the exponential term dominates over the prefactor $\sqrt{2/\pi \mathrm{N}}$. In this regime, based on our density functional theory (DFT) calculations, state II is more likely than state I when

$${}^{\Delta V_0}\!/_{2} - k_B T \ln\sqrt{{}^{23}\!/_{12}} < 0, \text{ i.e., } \Delta V_0 < 2 k_B T \ln\sqrt{{}^{23}\!/_{12}}$$

In our *ab initio* calculations, we considered several relative arrangements of the two bIm linkers within a unit cell (see Fig. SI35) and found that, in all but one arrangement, ΔV <0. Within the approximations leading to Eq. (3) and accuracy of the *ab initio* calculations, this indicates that bIm linkers favor state II over the uniform state I.

Table SI3. Difference in potential energy between the two representative minima R0 associated with states I and II, for several arrangements of the two bIm linkers within a unit cell.

	ΔV_0 / eV	$\Delta V_0/k_B T$
$V\left(R^0_{II_1}\right) - V\left(R^0_I\right)$	-0.2730	-10.92
$V\left(R^0_{II_2}\right) - V\left(R^0_I\right)$	-0.2301	-9.20
$V\left(R^0_{II_3}\right) - V\left(R^0_I\right)$	-0.0552	-2.21
$V\left(R^0_{II_4}\right) - V\left(R^0_I\right)$	0.2269	9.08

We summarize here the ansatz and approximations underlying Eq. (2). In Eq. (1), Ω_{I} and Ω_{II} are regions of the configuration space $\mathbb{R}^{3\mathrm{n}}$ (n: number of atoms) corresponding to states I and II. Specifically, I contains one bIm linker per unit cell, while II contains two bIm linkers per unit cell in half of the unit cells and none in the other half. Direct evaluation of this integral for a ~500 nm crystallite (our typical size), i.e., a sample containing ~ 10^9 atoms, is computationally infeasible. We therefore introduce approximations to obtain a tractable form of Eq. (1).

- First, we decompose Ω_{X}as $\Omega_{\mathrm{X}} = \sqcup_{i \in N_X} \Omega^i_{\mathrm{X}}$, where $X \in \{I, II\}$ and Ω^i_{X} is the region of configuration space associated with a specific arrangement of linkers. The exact boundaries of the regions Ω^i_{X} are not unique and are, to some extent, arbitrary. However, because linkers do not freely diffuse within the crystallite, configurations with non-negligible probability can be unambiguously assigned to one region Ω^i_{X} rather than another. With this partition, the configurational integral over Ω_X can be written as a sum over domains:
$$\int_{\Omega_{\mathrm{X}}} dR\, exp(-\beta V(R)) = \sum_{i \in \mathbb{N}_X} \int_{\Omega^{\mathrm{i}}_{\mathrm{X}}} dR\, exp(-\beta V(R)) \qquad (3)$$

This step does not reduce the dimensionality of the integral (it remains 3n-dimensional); it simply restricts the integration to smaller subdomains, at the price of introducing a sum over the distinct linker arrangements.

- The second step consists in grouping the degrees of freedom of Ω_X^i by unit cell. We denote by R^j (or, equivalently, by $\Omega_X^{i,j}$) the subset of coordinates associated with unit cell j, so that $R = (R^1,..., R^N)$.
- Third, we make the ansatz that $V(R) = \sum_{j\in N} V_j(R_j)$, where $V_j(R_j)$ is the potential energy associated to the atoms in the unit cell j of the crystallite. This ansatz amounts to assume that atoms in the cell j do not interact with atoms in the neighboring cells, which we know is false. Nevertheless, this ansatz allows us the express $\int_{\Omega_X^i} dR\, exp(-\beta V(R)) = \prod_{j=1,n} \int_{\Omega_X^{i,j}} dR\, exp(-\beta V_j(R))$. Indeed, $V_j(\cdot)$ can be envisaged as an effective potential in the mean field generated by the atoms in the neighboring cells. Nevertheless, we refrain to excessively formalize our model as our objective is assessing whether it is likely or not that the linkers are uniformly distributed.

Summarizing, within the approximations introduced above,

$$\int_{\Omega_X} dR\, exp(-\beta V_j(R)) = \sum_{i\in\mathbb{N}_X} \prod_{j=1,N} \int_{\Omega_X^{i,j}} dR\, exp(-\beta V_j(R)) \qquad (4)$$

Despite these simplifications, a direct evaluation remains intractable: one would still need to compute on the order of ~10^7 integrals, each in a very high-dimensional space (of order ~$10^2 - 10^3$degrees of freedom per unit cell). We further reduce the problem by i) retaining only one (or a few) metastable arrangements and by assuming that the per-cell integral $\int_{\Omega_X^{i,j}} dR\, exp(-\beta V_j(R))$ takes the same value for all relevant cell configurations; and ii) under this assumption, replacing the sum over $i \in \mathbb{N}_X$ by the corresponding multiplicity (degeneracy) of the cell configuration, which can be evaluated explicitly. Finally, iii) we evaluate the per-cell integral using the Laplace method (saddle-point approximation). This amounts to expanding $V_j(R_j)$ to second order around R_j^0, the minimum-energy configuration of the selected metastable state: $V_j(R_j) \sim V_j(R_j^0) + \frac{1}{2}(R_j - R_j^0)^T \hat{H}(R_j^0)(R_j - R_j^0)$, where $\hat{H}(R_j^0)$ is the Hessian matrix of the potential energy, with elements $\hat{H}_{\alpha\beta}(R_j^0) = \partial^2 V_j(R_j)/\partial R_{j\alpha}^0 \partial R_{j\beta}^0|_{R_j=R_j^0}$. Within this approximation, $\int_{\Omega_X^{i,j}} dR\, exp(-\beta V_j(R))$ reduces to a product of one-dimensional Gaussian integrals and can be written (up to a constant prefactor) as

$$\int_{\Omega_X^{i,j}} dR\, exp(-\beta V_j(R)) \propto exp(-\beta V_j(R_j^0)) \prod_{k=1,3n_{cell}-3} \sqrt{2\pi/\beta\lambda_k}, \qquad (5)$$

where $\lambda_k$is the kth eigenvalue of $\hat{H}(R_j^0)$. The product runs over $3n_{cell} - 3$ terms because the three translational modes correspond to zero-frequency (zero-curvature) modes. In periodic systems, rotations of the unit cell are not zero modes and therefore contribute as genuine vibrational modes. In practice, translational modes are treated

separately before performing the Gaussian integral: along each translational direction the quadratic term vanishes, so the integrand is unity and the corresponding contribution is simply the unit-cell length in that direction. These translational factors are identical for states I and II; hence they cancel in the ratio entering Eqs. (2) and (3).

In Eq. (5), contributions from linker arrangements that are equivalent by symmetry are accounted for through their multiplicity. This multiplicity reflects i) the number of symmetry-equivalent placements/orientations of the linker within a unit cell (e.g., the 24 equivalent arrangements of a single bIm in a unit cell) and ii) the number of distinct ways to distribute unit cells with different bIm content throughout the crystallite.

Despite the simplifications enabled by the manipulations and approximations introduced above, the problem remains formidable. We therefore further simplify the analysis by retaining only the lowest-energy metastable state for cases I and II. When one metastable state lies sufficiently lower in energy than all others, it dominates the corresponding configurational integral in Eqs. (1) and (3) through its Boltzmann weight. Within this single-metastable-state approximation per case (I and II), and accounting for the multiplicities of cases I and II, Eq. (1) reduces to Eq. (2).

In deriving Eq. (2), we additionally assume that the vibrational prefactor $\prod_{k=1,3n_{cell}-3} \sqrt{2\pi/\beta\lambda_k}$ is approximately the same for cases I and II, so that it cancels in the ratio. This cancellation is not exact, since the vibrational spectra (and thus the Hessian eigenvalues λ_k) of the two cases generally differ. However, the resulting prefactor ratio is expected to have a smaller impact than the ratio of the exponential terms, which scales with the number of unit cells in the crystallite (due to the product $P_{j=1,N}$).

Data reported in Table SI3 have been obtained from geometry optimizations of the unit cell of ZIFs under consideration (ZIF-8, ZIF-7-8 with 1 bIm linker, and ZIF-7-8 with 2 bIm linkers). These were performed within Density Functional Theory using the PBE exchange-correlation functional and the Projector-Augmented Wave (PAW) method [7], as implemented in the VASP package [8], [9], [10], [11]. Van der Waals interactions were accounted for using the DFT-D3 method with Becke–Johnson damping [12]. A plane-wave kinetic energy cutoff of 400 eV was employed.

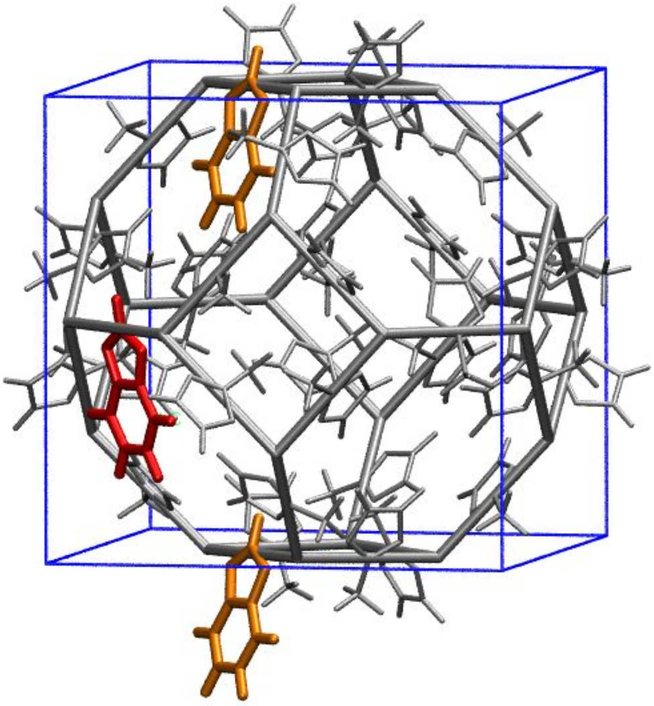

Figure SI35. Representation of the ZIF-7-8 unit cell (the most stable configuration) after geometric minimization via DFT. In this representation, all mIm linkers are displayed in gray. Two bIm are highlighted in red and orange colors (the double presence of the orange bIm linker is because of periodic boundary conditions).

Bibliography

[1] J. A. Thompson *et al.*, “Hybrid zeolitic imidazolate frameworks: Controlling framework porosity and functionality by mixed-linker synthesis,” *Chemistry of Materials*, vol. 24, no. 10, pp. 1930–1936, May 2012, doi: 10.1021/cm3006953.

[2] S. Berens, C. Chmelik, F. Hillman, J. Kärger, H. K. Jeong, and S. Vasenkov, “Ethane diffusion in mixed linker zeolitic imidazolate framework-7-8 by pulsed field gradient NMR in combination with single crystal IR microscopy,” *Physical Chemistry Chemical Physics*, vol. 20, no. 37, pp. 23967–23975, 2018, doi: 10.1039/c8cp04889d.

[3] J. Landers, G. Y. Gor, and A. V. Neimark, “Density functional theory methods for characterization of porous materials,” *Colloids Surf. A Physicochem. Eng. Asp.*, vol. 437, pp. 3–32, 2013, doi: 10.1016/j.colsurfa.2013.01.007.

[4] M. Thommes *et al.*, “Physisorption of gases, with special reference to the evaluation of surface area and pore size distribution (IUPAC Technical Report),” *Pure and Applied Chemistry*, vol. 87, no. 9–10, pp. 1051–1069, Oct. 2015, doi: 10.1515/pac-2014-1117.

[5] J. A. Thompson *et al.*, “Hybrid zeolitic imidazolate frameworks: Controlling framework porosity and functionality by mixed-linker synthesis,” *Chemistry of Materials*, vol. 24, no. 10, pp. 1930–1936, May 2012, doi: 10.1021/cm3006953.

[6] L. Guillemot, T. Biben, A. Galarneau, G. Vigier, and É. Charlaix, “Activated drying in hydrophobic nanopores and the line tension of water,” *Proc. Natl. Acad. Sci. U. S. A.*, vol. 109, no. 48, pp. 19557–19562, Nov. 2012, doi: 10.1073/pnas.1207658109.

[7] P. E. Blochl, “Projector augmented-+rave method.”

[8] G. Kresse and J. Furthmü, “Efficient iterative schemes for ab initio total-energy calculations using a plane-wave basis set,” 1996.

[9] G. Kresse and J. " Furthmiiller, “Efficiency of ab-initio total energy calculations for metals and semiconductors using a plane-wave basis set,” 1996.

[10] G. Kresse and J. Hafner, “Ab. initio molecular dynamics for liquid metals.”

[11] G. Kresse, “Ab initio molecular-dynamics simulation of the liquid-metal-amorphous-semiconductor transition in germanium.”

[12] S. Grimme, S. Ehrlich, and L. Goerigk, “Effect of the damping function in dispersion corrected density functional theory,” *J. Comput. Chem.*, vol. 32, no. 7, pp. 1456–1465, May 2011, doi: 10.1002/jcc.21759.
